# Solar‐Driven Ammonia Synthesis From Nitrate Reduction Paired With CO_2_ Capture for Sustainable Agriculture via a Robust CuPd Heterojunction

**DOI:** 10.1002/anie.9278631

**Published:** 2026-05-02

**Authors:** Weihua Guo, Xiaofeng Huang, Yangbo Ma, Yun Song, Zihao Li, Qiang Zhang, Yinger Xin, Jianjun Su, Mingming He, Ruixuan Wang, Rui Xue, Xing Li, Shibo Xi, Jian Wang, Shenlong Zhao, Siwei Zhang, Ben Zhong Tang, Alex K. Y. Jen, Ruquan Ye

**Affiliations:** ^1^ Department of Chemistry, State Key Laboratory of Marine Environmental Health City University of Hong Kong Hong Kong China; ^2^ City University of Hong Kong Shenzhen Research Institute Shenzhen Guangdong China; ^3^ Department of Materials Science and Engineering City University of Hong Kong Hong Kong China; ^4^ Department of Electrical and Computer Engineering University of Toronto Toronto Canada; ^5^ Institute of Chemical and Engineering Sciences A*STAR Singapore; ^6^ School of Energy and Environment City University of Hong Kong Hong Kong China; ^7^ CAS Key Laboratory of Nanosystem and Hierarchical Fabrication, CAS Center for Excellence in Nanoscience National Center for Nanoscience and Technology Beijing P. R. China; ^8^ Department of Chemistry and the Hong Kong Branch of Chinese National Engineering Research Center for Tissue Restoration and Reconstruction The Hong Kong University of Science and Technology Hong Kong China; ^9^ Guangdong Basic Research Center of Excellence for Aggregate Science, School of Science and Engineering The Chinese University of Hong Kong Shenzhen (CUHK‐Shenzhen) Guangdong China

**Keywords:** amorphous/crystalline interfaces, carbon capture, crop yields, nitrate reduction, Solar‐driven

## Abstract

The transition to sustainable agriculture requires technologies that simultaneously enhance crop yields and reduce environmental impacts. Solar‐driven nitrate valorization, when coupled with CO_2_ capture from industrial flue gas, presents a promising dual strategy for producing high‐value fertilizers while mitigating carbon emissions. However, its practical implementation is hindered by two interrelated challenges: (i) the intermittent nature of solar irradiation and (ii) the competitive hydrogen evolution reaction (HER), which severely compromises Faradaic efficiency (FE) of desired nitrogenous products. Here, we address these challenges by designing a heterogeneous CuPd electrocatalyst featuring an amorphous/crystalline heterojunction. This catalyst suppresses HER across a broad potential window (−0.4 to −1.4 V), maintaining >80% FE(ammonia) for >100 h. The catalytic robustness enables stable solar‐powered electrolysis even under low irradiation (0.4 sun), achieving >70% FE(ammonia) and 6% solar‐to‐fuel conversion efficiency, while catholyte simultaneously captures CO_2_ at a rate of 6–20 mg h^−1^. Techno‐economic analysis demonstrates cost competitiveness against biological counterparts. When applied to plant cultivation, this artificial photosynthesis system boosts solar‐to‐biomass conversion efficiency by 3.5‐fold compared to natural photosynthesis. By unifying solar energy harvesting, waste nitrate reduction, and carbon sequestration, our work provides a scalable blueprint for a closed‐loop agrochemical ecosystem and advanced catalyst design for intermittent renewable‐powered electrosynthesis.

## Graphical abstract

Laser‐assisted synthesis of CuPd amorphous/crystalline heterojunction demonstrates >80% Faradaic efficiency for nitrate‐to‐ammonia electroreduction across wide potential windows. The integrated solar‐powered electrolyzer achieves 6% solar‐to‐ammonia energy efficiency at 0.4 sun, 6–20 mg h^−1^ CO_2_ capture rate, and stability exceeding 100 h. This artificial photosynthesis system boosts solar‐to‐biomass conversion efficiency by 3.5‐fold compared to natural photosynthesis.

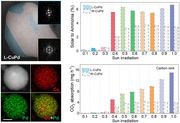

## 1 Introduction

The urgent need for sustainable agricultural practices calls for innovative solutions that boost crop productivity while reducing environmental harm. Solar energy, as a renewable and abundant resource, offers a viable pathway to drive chemical processes such as fertilizer synthesis [1, 2, 3]. Solar‐driven carbon‐negative nitrate valorization is a particularly promising strategy, harnessing sunlight to convert nitrogen waste into value‐added ammonia or nitrate fertilizers [4, 5, 6, 7, 8, 9]. This approach not only supports sustainable agriculture but also enables carbon sequestration, delivering dual environmental benefits [10, 11, 12]. However, the practical implementation of solar‐driven nitrate valorization faces major challenges stemming from the inherent variability of solar irradiance. Diurnal cycles, weather conditions, and seasonal variations lead to fluctuations in energy input, resulting in voltage attenuation and downgraded process efficiency and stability. Additionally, the competitive hydrogen evolution reaction (HER) further lowers the Faradaic efficiency of the desired nitrogen products, particularly under variable power input conditions.

The instability of solar irradiance poses critical operational challenges, particularly in maintaining steady voltage and power output in solar‐driven electrochemical systems [13]. To scale solar‐driven technologies from laboratory research to industrial applications, two key challenges must be addressed [14]. First, high‐performance electrocatalysts should be developed to achieve industrially relevant nitrate production rates while maintaining high selectivity [15, 16, 17]. Second, durable catalysts should be designed to resist degradation under prolonged solar irradiation and harsh electrochemical conditions [18, 19, 20]. A major bottleneck in these challenges is the competing HER, which dominates at higher applied voltages, reducing the faradaic efficiency (FE) for nitrate reduction [21, 22, 23, 24]. Thus, engineering catalysts that selectively suppress HER while maintaining high activity for nitrate‐to‐ammonia conversion is critical for maximizing solar‐powered wastewater valorization.

**FIGURE 1:**
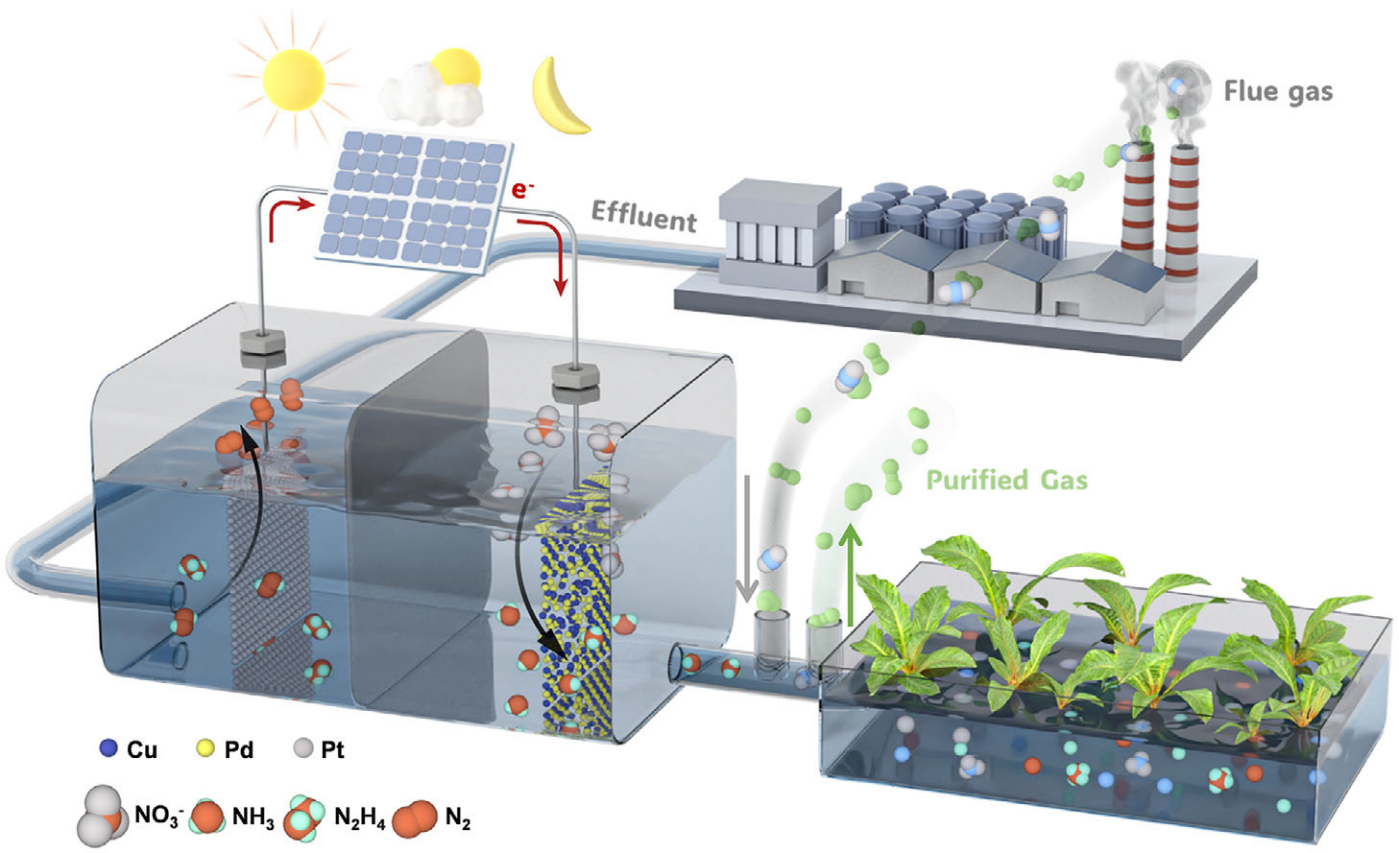
Schematic of Artificial photosynthesis. Flue gases and effluent from factories are converted into plants assisted by solar cells‐driven electrochemical cell.

We have innovatively introduced an artificial solar energy system that couples anodic hydrazine hydrate oxidation with cathodic nitrate reduction to sustainably convert two of the three major waste streams from industrial processes—nitrate‐containing and carbon dioxide‐rich flue gases—into nutrients for plant growth (Figure 1). Powered by solar cells, this integrated system oxidizes hydrazine hydrate wastewater to nitrogen gas, electrochemically reduces aqueous nitrate (NO_3_^−^) to ammonium (NH_4_^+^), and simultaneously absorbs carbon dioxide from the flue gas, thereby addressing water pollution and greenhouse gas emissions while producing valuable nitrogen and carbon compounds.

To address the voltage instability, designing catalysts that work efficiently across a wide potential is imperative. Among various strategies such as formation of alloy [25, 26, 27, 28, 29], single atom [21, 22, 23, 30, 31, 32, 33], and two‐dimensional material [34], we here leverage amorphous/crystalline heterostructures to design our catalysts [35, 36, 37, 38], which has successfully tailored the catalytic activity in diverse applications such as CO_2_ reduction and water splitting [37, 39, 40, 41]. Specifically, we report a facile laser‐based method to synthesize a mixed‐phase CuPd catalyst for efficient solar‐driven ammonia production from nitrogenous wastewater. The engineered amorphous‐crystalline interfaces optimize intermediate adsorption while suppressing HER, enabling stable operation under fluctuating solar conditions. The catalyst retains an ammonia FE of exceeding 70% across an extensive potential window of −0.2 to −1.4 V. Such superior performance allows solar‐powered electrolysis to operate steadily under low light conditions of 0.4 sun, achieving over 70% FE_ammonia_ and a 6% solar‐to‐fuel conversion efficiency, while CuPd synthesized from wet‐chemical method only shows 2% of solar‐to‐fuel conversion efficiency. Furthermore, the catholyte exhibits a carbon dioxide uptake capacity ranging from 6 to 20 mg·h^−^^1^, which is almost twice‐three folds of that by wet‐chemical one. This study bridges the gap between fundamental electrocatalyst design and process engineering, offering a scalable solution for solar‐powered nitrate valorization. By addressing voltage attenuation and catalytic selectivity, we advance the practical deployment of carbon‐negative technologies in sustainable agriculture.

## 2 Results and Discussion

### 2.1 The Synthesis and Performance of Catalysts

**FIGURE 2:**
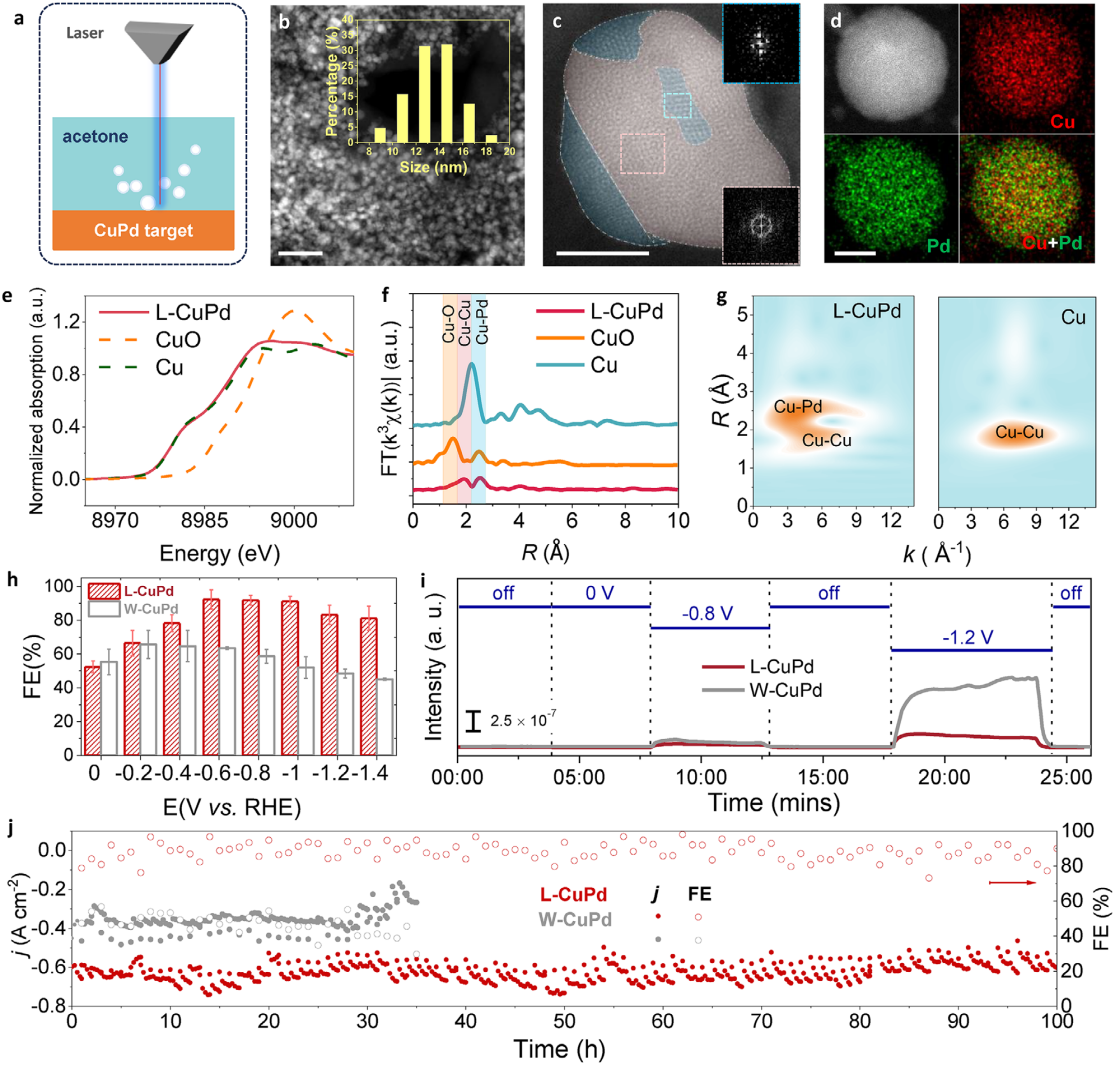
Morphological characterization, structural characterization, and electrochemical NITRR performance of L‐CuPd. a) A schematic of laser processing. b) STEM, c) Spherical aberration‐corrected HAADF‐STEM image. The blue area represents amorphous CuPd, and the pink area is crystalline CuPd. d) corresponding EDX mapping of L‐CuPd. e) Normalized XANES, f Fourier transformed EXAFS and g) WT–EXAFS Cu K‐edge spectra of L‐CuPd, L‐Cu_9_Pd_1_, Cu and CuO. h) Faraday efficiency of L‐CuPd and W‐CuPd in different potentials. i) Signal of H_2_ in online DEMS over L‐CuPd and W‐CuPd during different potentials. j) Stability test of L‐CuPd and W‐CuPd at ‐1.2 V in 1 M KNO_3_. Scale bar: 100 nm in (b), 5 nm in (c) and (d).

To maximize solar energy utilization in wastewater valorization, we introduce the laser to synthesize the mixed‐phase catalyst to meet the stringent catalytic requirements. Figure 2a depicts the synthesis of laser‐engineered CuPd nanoparticles. Transmission electron microscopy (TEM), high‐angle annular dark‐field scanning transmission electron microscopy (HAADF‐STEM), and energy‐dispersive x‐ray spectroscopy (EDX) elemental mapping were used for the characterization of morphology and structure of L‐CuPd (Figures S1–S4)_._ The heterogeneous CuPd exhibits uniform size, with an average size of approximately 13 nm (Figure 2b). The fast Fourier transforms (FFTs) of the selected area in Figure 2c shows the co‐existence of a typical diffusive amorphous halo structure (red region) and a crystalline state structure (blue region). The elemental mapping profiles reveal that Cu and Pd elements are distributed evenly (Figure 2d).

To further investigate the local chemical structural change of L‐CuPd, XAS analysis was carried out at the Cu K edge. As shown in Figure 2e, x‐ray absorption near‐edge structure (XANES) of L‐CuPd is similar to that of Cu, which suggests the valence of Cu is close to 0 in L‐CuPd [42]. The Fourier transformed extended x‐ray absorption fine structure (EXAFS) shows that L‐CuPd has an additional Cu‐Pd path at *R* = 2.5 with similar intensity to Cu–Cu, which indicates that the Cu is primarily connected to Cu and Pd atoms in L‐CuPd, consistent with the homogeneous elemental distribution in the EDX mapping results (Figures 2f and S3–S7; Table S1) [43]. The wavelet transform is used to further visualize the localized coordination environments (Figure 2g). The wavelet transform diagram also clearly shows the formation of Cu─Pd scattering path in L‐CuPd, which differs distinctly from that of Cu with Cu─Cu bonds [43]. The above characteristics show that the L‐CuPd is a metallic state with uniform mixed phases [39].

We compare the nitrate electroreduction (NITRR) catalytic performance of L‐CuPd to W‐CuPd (W‐CuPd nanoparticles synthesized by wet‐chemical method, see method and Figures S8–S10) in 1 M KOH and 1 M NO_3_^−^. The concentrations of the products, including nitrite (NO_2_^−^) and NH_3_, produced after NITRR were analyzed (Figures S11–S18). As shown in Figure 2h, the L‐CuPd has a higher ammonia FE of above 80% in a wide potential window from −0.4 to −1.4 V vs. RHE (Figure 2h). In comparison, W‐CuPd only achieves high FEs at potentials below −0.4 V vs. RHE. To elucidate the performance differences between L‐CuPd and W‐CuPd, in situ differential electrochemical mass spectrometry (DEMS) was carried out during NITRR (Figure 2i). The results reveal that W‐CuPd exhibits a stronger H_2_ signal than L‐CuPd, particularly at −1.2 V. Careful structural comparison suggests that the higher crystalline CuPd content in W‐CuPd (W‐CuPd, crystalline vs L‐CuPd, amorphous/crystalline) is primarily responsible for its higher Faradaic efficiency (FE) for H_2_ and the correspondingly lower FE for ammonia, consistent with previous reports. This is mainly because crystalline CuPd is prone to hydrogen production. The result of differential electrochemical mass spectrometry (DEMS) during NITRR in different potentials also proves it. It is clearer that the intensity of H_2_ of W‐CuPd is higher than that in L‐CuPd, especially at −1.2 V (Figure 2i). In addition, the L‐CuPd electrocatalyst exhibits significantly enhanced long‐term stability, maintaining consistent performance for approximately 100 h—nearly three times longer than that of the W‐CuPd (35 h) (Figure 2j). This exceptional durability effectively meets the stringent stability requirements for practical applications under real operating conditions. Post‐structural characterization shows that L‐CuPd maintains the spherical structure after electrolysis (Figures S19–S25). The enhanced stability primarily stems from the unique amorphous/crystal structure of L‐CuPd, which enables the materials to self‐adjust and resist structural disruptions throughout electrocatalysis [36, 44].

### 2.2 Solar‐Assisted Nitrogen Conversion and Carbon Fixation System

**FIGURE 3:**
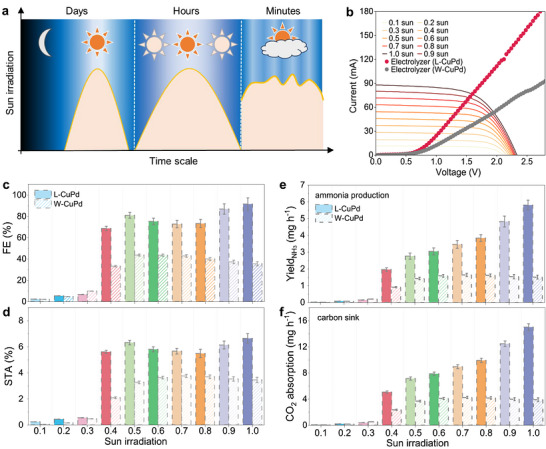
Solar‐assisted nitrogen conversion and carbon fixation system. a) Scheme showing solar irradiation in different conditions. b) Intersections of JV characteristics of perovskite solar cells at different sun with the load curve of an electrochemical cell of L‐CuPd and W‐CuPd. c) Ammonia selectivity, d) solar‐to‐ammonia efficiency, e) yield, and f) CO_2_ adsorption capacity at different solar intensities of L‐CuPd and W‐CuPd.Error bars represent the standard deviation of three independent measurements.

Building on the excellent performance of L‐CuPd, we then construct a solar‐driven electrolyzer to produce ammonia fertilizer from nitrate, which is subsequently used to absorb carbon dioxide and cultivate plants. To achieve optimal energy utilization, the voltage and current of the solar cell need to match those in the electrochemical system. However, challenges arise when: (1) The intensity of sunlight varies at different times of the day, resulting in changes in the efficiency of solar power generation (Figure 3a). (2) The solar cell's maximum power point (MPP) mismatches that of the electrochemical system due to solar fluctuation, resulting in wasted solar energy (Figure 3b). Therefore, catalysts must maintain high NITRR performance over a wide potential window to improve solar‐to‐fuel conversion efficiency.

In a typical solar‐powered electrolysis system, the anode reaction adopts oxygen evolution reaction (OER). However, OER has inherent disadvantages, including the high theoretical potential (1.23 V) and slow kinetics. Thus, a cell voltage of 1.6–2.0 V will be required, resulting in large energy consumption from water oxidation [45, 46, 47]. To reduce energy consumption, replacing OER with small molecules oxidation at low potential is necessary. Studies have found that hydrazine electrooxidation (HzOR), which has a low thermodynamic onset potential, is a promising alternative to HzOR for energy‐saving ammonia production [48, 49]. Additionally, this process of anodically oxidizing toxic hydrazine (N_2_H_4_) wastewater and converting it into harmless nitrogen gas (N_2_) achieves a “kill two birds with one stone” outcome: it not only treats the toxic pollutant but also provides the necessary electrons and reaction conditions for ammonia synthesis [45, 50, 51]. It represents a typical “waste‐treating‐waste” green chemistry strategy. Thus, pairing wastewater treatment containing hydrazine with NITRR will be a feasible and environmentally friendly pathway (Figures S26–S33).

The solar‐to‐ammonia cell voltage (*V*) can be divided into four parts:

$$V=E+\eta_{\mathrm{NITRR}}+\eta_{\mathrm{HzOR}}+\mathrm{iR}$$

where *E* symbolizes the standard thermodynamic cell potential for the conversion of nitrate and hydrazine to ammonia and N_2_, *η_NITRR_* and *η_HzOR_* indicate the overpotentials for ammonia formation and HzOR, and iR signifies the ohmic loss between two electrodes.

To maximize solar energy utilization near the maximum power point (MPP), we employed two parallel‐connected perovskite solar cells fabricated with optimized perovskite active layers and encapsulation strategie [52]. This configuration ensures stable power output under ambient conditions to drive the electrolyzer containing 50 mM KNO_3_ + 1 M KOH. We also compare the performance of L‐CuPd with W‐CuPd (Figures S34–S38). As shown in Figure 3b, the *J–V* curves of solar panels change with sunlight, resulting in a shift of MPP. The peak photovoltaic solar‐to‐electricity conversion efficiency can reach its maximum at 1.6 V and 76.6 mA (PCE = 128 mW) for L‐CuPd, and 1.9 V and 53.2 mA (PCE = 101 mW) for W‐CuPd at 1 sun, respectively. We simulate and test the experiment of electroreduction of nitrate to ammonia driven by solar cells under different solar intensities, as shown in Figure 3c,d. Figure 3c demonstrates a positive correlation between solar irradiance intensity and selectivity. Under 0.4‐sun illumination, the system achieves an FE of 70% for nitrate reduction to ammonia, with a corresponding solar‐to‐ammonia (STA) conversion efficiency of 6%. As irradiance increases beyond this threshold, the system maintains ammonia conversion efficiencies exceeding 75% while preserving STA efficiency at approximately 6% across the tested irradiance range. In comparison, the FE of W‐CuPd reaches its highest of 43% at 0.5 sun, which gradually decreases to 35% at 1 sun. The STA of W‐CuPd only reaches ∼3.6% at >0.5 sun (Figure 3d). This is due to the severe hydrogen evolution for W‐CuPd.

We further characterized the two catalysts' ammonia production and carbon fixation capabilities under different solar radiation intensities. As shown in Figure 3e, quantitative analysis reveals a linear relationship between irradiance intensity (0.4–1.0 sun) and ammonia production rate, yielding 2∼6 mg·h^−1^ of NH_3_, which is 2–5 folds of ammonia production rate of W‐CuPd. The ammonia solution generated simultaneously can be used for carbon dioxide capture from flue gas [12, 53]. Under different solar intensities, L‐CuPd efficiently captures carbon dioxide from flue gas at a rate of 5–15 mg·h^−1^ for pH ∼7 electrolyte (Figure 3f) and 6–20 mg·h^−1^ for pH 14 electrolyte (Figure S39), which are twice the carbon dioxide adsorption capacity of W‐CuPd. The resulting ammonia solution demonstrates remarkable dual functionality, which could enable pH modulation to optimal ranges (pH 7.5–8.5) for agricultural applications. This integrated process achieves simultaneous carbon sequestration and nutrient solution conditioning, as evidenced in Figure 3e,f. Notably, the carbon capture mechanism primarily involves the formation of ammonium bicarbonate (NH_4_HCO_3_), a stable compound that serves as both a carbon sink and a fertilizer. The experimental results demonstrate that, owing to its superior electrochemical performance, L‐CuPd achieves an ammonia Faradaic efficiency of 70% even under fluctuating solar irradiation conditions at 0.4 sun, along with remarkable ammonia production and carbon fixation performance, which is two to three times higher than that of W‐CuPd. This significantly enhances the overall efficiency and practical feasibility of the solar‐driven ammonia and carbon fixation system.

### 2.3 Scalable Solar‐Boosted Plant Growth by Artificial Photosynthesis

**FIGURE 4:**
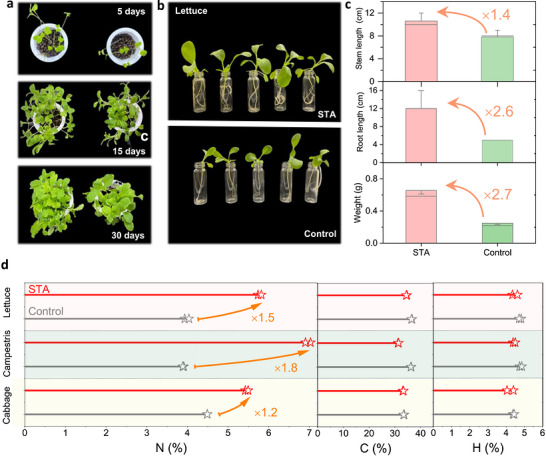
Scalable solar‐boosted plant growth by artificial photosynthesis. a) Picture of solar driven electrocatalyst NITRR to produce ammonia for lettice. b) Plant growth at different stages in STA and control group. c) Comparison of plants in the STA and control group. Stem length range, root length range, and weight in different groups. d) N, C, and H contain different vegetable parts under the STA and control groups.

Considering the outstanding solar energy conversion efficiency and carbon dioxide absorption capacity of L‐CuPd, the STA conversion has been successfully realized for hydroponic plant cultivation under natural daylight conditions (Figures 4a and S40–S44). The control groups were cultivated at the same manner, except using nitrate electrolyte without solar‐driven electrolysis. Comparative growth analyses demonstrate significantly enhanced plant development in the STA‐treated group when compared to the control group (Figure 4b). Following 45 days of growth, quantitative assessments reveal substantial improvements across all measured morphological parameters (Figure 4c). Specifically, the stem length of the STA group exhibits a 40% increase (3.8 ± 0.69 cm plant^−1^) compared to the control group (2.5 ± 0.5 cm plant^−1^). Root system development shows particularly striking enhancement, with STA‐treated plants achieving a root length of 9.8 ± 1.34 cm, which is 2.6‐fold greater than that of control specimens. Additionally, biomass accumulation in the STA group (2.36 ± 0.15 g plant^−1^) is significantly higher than that in the control group (2.01 ± 0.13 g plant^−1^).

We further investigated the nitrogen (N), carbon (C), and hydrogen (H) contents in vegetables of equal mass from both the STA‐treated and control groups (Figure 4d). Elemental analysis indicates enhanced nutrient assimilation in the STA‐treated plants, with significantly higher N content observed under comparable C and H levels compared to the control. Specifically, the N contents in lettuce, campestris, and cabbage after STA treatment were measured as 6.90 ± 0.62, 2.32 ± 0.26, and 8.60 ± 0.51 g plant^−1^, which are 1.5, 1.8, and 1.2 folds of that in the control group, respectively. All values reported for the STA group exceed those of the control, with the most prominent improvement observed in campestris. These results demonstrate that the solar‐electrosynthesized ammonia fertilizer can effectively enhance nitrogen content in plants and further promote plant growth.

### 2.4 Assessments of Agriculture Sysstem

**FIGURE 5:**
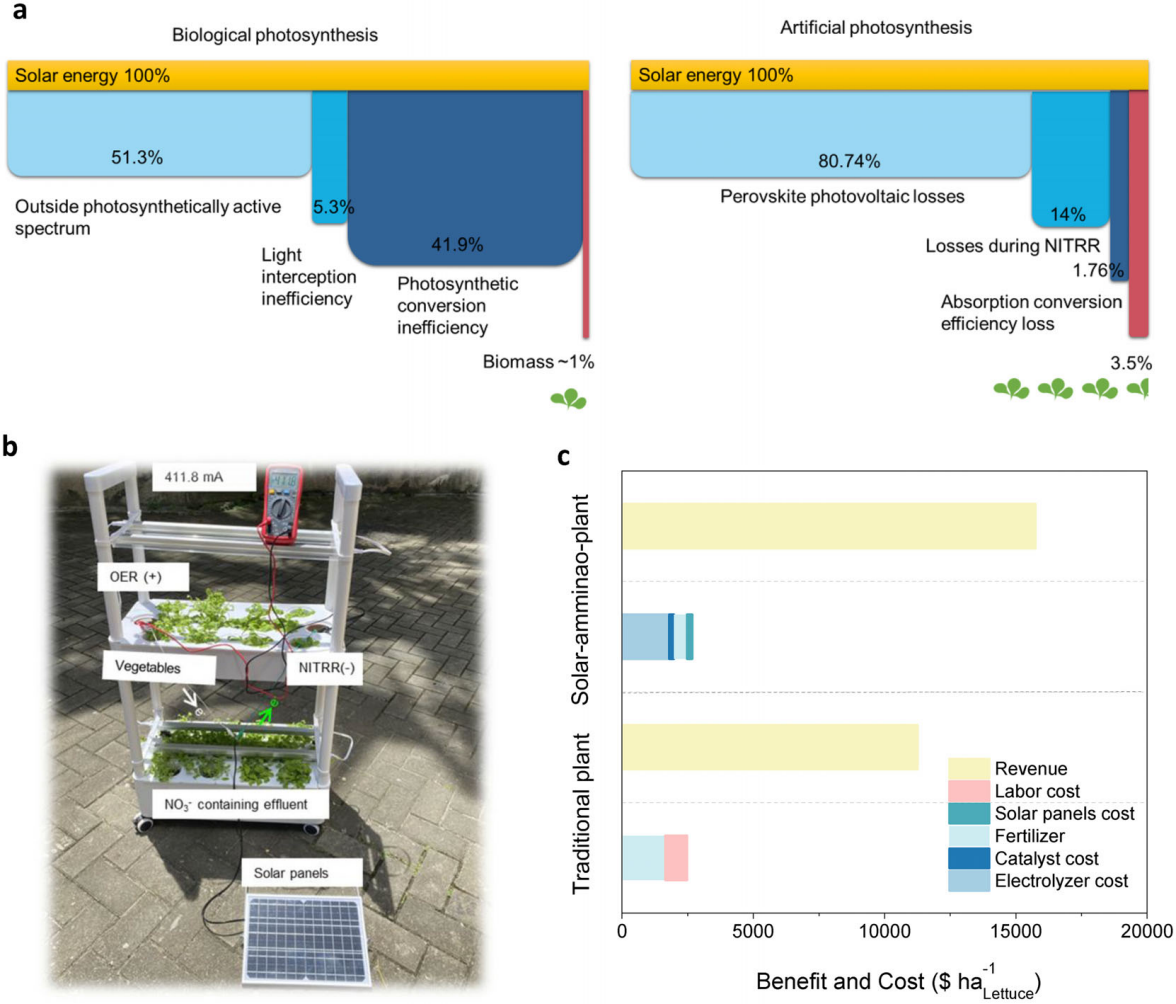
Analysis of application scenarios and assessments. a) Comparison of the efficiency of artificial and biological photosynthesis in converting solar energy into plants [2]. Blue illustrates the energy losses during the transformation from incident sunlight and red represents the energy to final plant. The efficiency values for biological photosynthesis are derived from the research conducted by Zhu et al., [55]. **b**) A solar‐to‐plant system for converting nitrate‐containing wastewater into ammonia for agriculture. It produces more than 400 mA of current under daylight. c) Benefit‐cost analysis of the proposed strategy and the traditional planting method.

We conducted a comparative analysis of artificial (our solar to biomass by integrated systems) and biological (natural process) photosynthesis in terms of energy conservation [2]. We first compare the solar‐to‐mass conversion efficiency by considering several energy transfer pathways (See Note S1 for estimation). Our findings reveal that the energy conversion pathway encompassing photovoltaic energy capture, electrolysis, ammonia synthesis, and subsequent utilization by plants exhibits a solar‐to‐biomass energy conversion efficiency of ∼3.5%, which is approximately 3.5 times higher than that of the natural biological photosynthesis process (Figure 5a). The above results indicate that the solar‐to‐ammonia‐to‐plant growth system has the potential for enhancing energy‐harvesting efficiency in agriculture.

We further compare the benefits and costs of the artificial and biological photosynthesis processes. As illustrated in Figure 5b, the system employs commercial solar panels to directly convert nitrate–laden wastewater into ammonia, serving as a nutrient source for plant growth. Under conditions of high‐intensity solar radiation, the generated current surpasses 400 mA (the value that can be further enhanced with improvements in solar cell performance). A cost‐effectiveness assessment comparing this approach to conventional growing methods show that solar‐driven ammonia production for plant growth has significant economic advantages over traditional cultivation techniques (Figure 5c and Note S2) [54]. Although the cost of the solar‐ammonia‐plant process is higher, it shows a higher revenue from faster plant growth. In addition, this process can simultaneously capture carbon dioxide, which further contributes to environmental conservation.

## 3 Discussion

In summary, this study proposes a sustainable agricultural approach that integrates solar‐driven nitrate valorization with wastewater treatment. The designed heterogeneous CuPd electrocatalyst effectively overcomes key technical challenges associated with fluctuating solar irradiance and competitive reactions, enabling efficient and stable ammonia synthesis from wastewater. By coupling with the treatment of toxic hydrazine hydrate wastewater at the anode, a waste‐treating‐waste strategy is achieved, which successfully converts industrial nitrate‐ and hydrazine‐containing wastewater, along with carbon dioxide from exhaust gas, into plant nutrients under practical solar‐driven conditions, thereby promoting plant growth. This artificial photosynthesis system demonstrates an ammonia conversion efficiency 3.5 times higher than that of natural photosynthesis, with high selectivity, offering a feasible solution for renewable fertilizer production and significant carbon sequestration potential. Techno‐economic analysis further confirms its cost competitiveness compared to conventional methods, highlighting its scalability and practicality. This research paves the way for developing closed‐loop agricultural systems and provides a sustainable pathway toward carbon‐negative agriculture and transformative nitrogen management in the agricultural sector.

## 4 Experimental Section

### 4.1 Material Synthesis

#### 4.1.1 Preparation of L‐CuPd

To synthesize L‐CuPd, a pulsed Nd: YAG laser (Nimma‐600, Beamtech) was utilized to ablate a CuPd target submerged in acetone. The laser parameters were as follows: pulse width of 10 fs, wavelength of 1,064 nm, and pulse frequency of 15 Hz. The CuPd material used was 99.99% purity and was shaped into a rectangular target measuring 20*90 mm in length and 5 mm in thickness. The laser ablation process lasted for 30 min, resulting in the formation of a solution containing CuPd. This solution was then centrifuged at 13,320 x g for 15 min and repeated thrice to enrich the CuPd content.

#### 4.1.2 Preparation of W‐CuPd

First, 5 mg Cu(acac)_2_, 5 mg Pd(acac)_2_, 20 mL oleylamine, and 4 mL 1,2 butanediol were added to a 50 mL round‐bottom flask, and then the mixture was heated at 180° for 5 h. After cooling, it was divided into two tubes and centrifuged three times at 13,320 x g for 15 min.

#### 4.1.3 Characterizations

The high‐angle annular dark‐field scanning transmission electron microscopy (HAADF‐STEM) technique, in combination with energy‐dispersive x‐ray spectroscopy (EDX), was utilized to capture STEM and mapping images at an accelerating voltage of 200 kV. X‐ray diffraction (XRD) patterns of the samples were obtained using a Bruker D2 instrument with a Cu Kα source. The scan step was set at 10° min^−1^, covering a range between 10° and 80°. X‐ray photoelectron spectroscopy (XPS) data were collected using a Thermo ESCALAB 250Xi spectrometer equipped with a monochromatic Al Kα radiation source (1486.6 eV) and a pass energy of 20.0 eV. The data were calibrated using the C 1s peak at 284.8 eV. X‐ray absorption fine structure (XAFS) measurements were conducted in transmission mode at the x‐ray absorption fine structure for catalysis at the beamline of the Singapore Synchrotron Light Source. The synchrotron operated at 700 MeV with a beam current of 200 mA. Data processing was carried out using the Athena and Artemis software packages.

## 5 Electrochemical Measurements

### 5.1 Electrochemical Reduction of Potassium Nitrate in an H‐cell

All NITRR experiments were conducted utilizing a three‐electrode setup in a dual‐compartment H‐cell, which was divided by an ion‐exchange membrane (Nafion 117) and linked to a CHI 650 electrochemical workstation (Chenhua, Shanghai). The prepared L‐CuPd was loaded onto 0.5 cm^2^ carbon paper, Hg/HgO, and a platinum plate, which served as the working electrode, reference electrode, and counter electrode. A 15 mL mixture of KOH/KNO_3_ solution (in varying configurations) was employed as the cathode and anode electrolyte. All potentials were measured against the reversible hydrogen electrode (RHE) using the formula ERHE = E_Hg/HgO_ +0.0591*pH + 0.098. Cyclic voltammetry (CV) and LSV were conducted at scan speeds of 10 and 5 mV s^−1^, respectively. Potentiostatic tests were performed at various potentials for 3600 s at a stirring speed of 800 rpm. The potential range for calculating the NH_3_ FEs and yield rates ranged from 0 to −1.4 V vs. RHE, with intervals of −0.2 V. Isotopic labeling trials were conducted using the same techniques at −1.0 V vs. RHE, except that the nitrogen source was replaced with 99% ^15^NO_3_^−^. Unless otherwise stated, all tests were conducted in an environmental chamber at room temperature without iR‐compensation.

### 5.2 Product Detection

The gas and liquid products under different potentials during NITRR through UV–vis spectrophotometry and nuclear magnetic resonance (NMR).

### 5.3 Ammonia Detection

^1^H nuclear magnetic resonance (^1^H NMR) was recorded on an AVANCE III HD 300 system to detect ammonia. The pH value of the final electrolyte was adjusted to be weakly acidic with 2 M HCl. Maleic acid (C_4_H_4_O_4_, 50 ppm) was employed as the external standard to calibrate the standard curve of NH_4_^+^ using the peak area ratio between NH_4_^+^ and maleic acid. The isotope labeling experiments were also measured using the same process.

### 5.4 Nitrite Detection

The concentration of nitrite was determined using UV–vis spectrophotometry following the standard procedure. Initially, a color reagent was prepared using a blend of p‐aminobenzene sulfonamide (4 g), N‐(1‐naphthyl) ethylenediamine dihydrochloride (0.2 g), deionized water (50 mL), and phosphoric acid (10 mL, *ρ* = 1.685 g ml^−1^). The electrolyte sample was then procured and diluted to fall within the detection range. Subsequently, 40 µl of the color reagent was combined with a 2.0 mL sample solution, mixed well, and left to rest for 20 min under normal conditions. The absorption intensity at a wavelength of 540 nm was subsequently measured using a UV–vis spectrophotometer (UV‐2600). A concentration‐absorbance curve was established using a series of standard potassium nitrite solutions that were linearly fitted and prepared in advance. Finally, the concentrations of the nitrite product were computed based on the tested absorbance and the established standard curve.

### 5.5 Hydrazine Detection

The concentration of hydrazine in the electrolyte was determined following the method established by Watt and Chrisp. A color reagent was first prepared by dissolving 5.99 g of 4‐(dimethylamino)benzaldehyde in a mixture of 30 mL of hydrochloric acid and 300 mL of ethanol. Subsequently, 2.5 mL of the catholyte was collected and combined with an equal volume of the freshly prepared reagent. The resulting mixture was incubated in the dark at ambient temperature for 20 min to allow full color development. The absorbance of the solution was then recorded at 457 nm using a UV–vis spectrophotometer (Shimadzu UV‐1700). To determine the hydrazine concentration, a calibration curve was constructed using standard solutions with known N_2_H_4_ concentrations, and the unknown concentration in the post‐electrolysis electrolyte was calculated accordingly.

### 5.6 Hydroxylamine Detection

Hydroxylamine generated during electrolysis was quantified via ^1^H NMR spectroscopy following an oximation reaction. In this procedure, NH_2_OH present in the catholyte was derivatized by reaction with an excess of glyoxylic acid (C_2_H_2_O_3_). Specifically, after 1 h of electrolysis, 0.4 mL of the catholyte was collected and mixed with 0.1 mL of deuterated dimethyl sulfoxide (DMSO‐d_6_) and 12.5 µL of an aqueous solution containing 50 wt.% glyoxylic acid. The resulting mixture was then subjected to NMR analysis for the identification and quantification of the corresponding oxime derivative.

The Faradaic efficiency (FE) and yield were calculated according to the following equations:

(1) $$FE_{liquid}=\frac{Q_{liquid}}{Q_{total}}\times 100\%=\frac{n_{liquid}\times N\times F}{j\times t}\times 100\%$$

(2) $$Yield_{NH3}=\frac{n_{NH3}\times M_{NH3}}{m_{Catalyst}\times t}$$

*n* is the amount of product (mol), *N* is the number of electron transfer to form a molecule of product; *F* = 95200 C mol^−1^ is Faraday constant, *T* = 298 K is the temperature (K) and *R* is the molar gas constant = 8.31 J (mol K) ^−1^, *j* is the total current, *t* is the electrolysis time (s), *M* is the Molar mass of product (g mol^−1^), *m* is the quality of catalyst (mg).

### 5.7 Computational Methods

The density functional theory (DFT) calculations were performed by the Vienna ab initio simulation package (VASP) [56]. Projector augmented wave (PAW) pseudopotential was used for the core electrons. The generalized gradient approximation (GGA) in the form of Perdew–Burke–Ernzerhof (PBE) was applied for the exchange correlation potentials [57]. Van der Waals (vdW) interaction was considered at the DFT‐D2 level as proposed by Grimme (IVDW = 12) [58].,The cutoff energy was 450 eV for the valence electrons. The atoms were relaxed fully until the energy convergence reached 0.00001 eV and the force acting on each atom was less than 0.02 eV/Å. The W‐CuPd alloy was obtained by replacing one Cu (mp‐30) atom with one Pd atom [59]. The CuPdO_2_^−^supported CuPd model was built by stacking the CuPd(111) surface on the CuPdO_2_ (mp‐996971) (111) surface as L‐CuPd(111). To make the L‐CuPd(111) model amorphous, the model was subjected to ab initio molecular dynamics (AIMD) calculations. AIMD simulations were run for 20 ps as equilibration with time steps of 2 fs, performing a constant temperature of 298 K in the Nosé–Hoover canonical ensemble. The Gibbs free energy change for each reaction step is calculated as,

$$\Delta G=\sum G\left(products\right)\;-\sum G\left(reactants\right)$$

where *G*(i) is the Gibbs free energy of species i. Gibbs free energy of each species was calculated as G = E+ZPE‐TS, where E is the total energy obtained from DFT calculations, ZPE is the zero‐point energy, and S is the entropy. Temperature T was set to be 298 K.

### 5.8 Practical Applications of the Solar‐to‐Ammonia Combined System

The integrated system was configured as a two‐compartment electrochemical cell separated by a cation exchange membrane (Nafion 117): the anode compartment featured a platinum electrode with 0.1 M hydrazine hydrate (N_2_H_4_·H_2_O) dissolved in 1 M KOH as the anolyte; the cathode compartment employed a carbon paper electrode loaded with the L‐CuPd catalyst with 50 mM KNO_3_ dissolved in 1 M KOH as the catholyte; and the Nafion 117 membrane served to prevent hydrazine crossover while allowing ionic conduction (K^+^ or OH^−^). This NITRR H‐cell configuration was powered by two perovskite solar cells connected in series. The PV‐EC system was operated under 1 sun or less than 1 sun illumination (AM 1.5G) using a solar simulator light source (Zolix, GLORIA‐X500A), with the light intensity calibrated by a Si reference (Zolix). The full‐cell voltage and current of the PV‐EC system were measured using a source meter (Keithley, 2612B). The following equation calculated the solar‐to‐ammonia conversion efficiency (STA):

(3) $$\;\eta_{STA}=\frac{P_{out}}{P_{in}}=\frac{I_{OP}\times E_{0}\times FE}{P_{light}\times A}$$

*I_OP_* is the operating current of NO_3_^−^‐to‐NH_3_, *E_0_* is the thermodynamic potential of NO_3_^−^‐to‐NH_3_ (0.69 V vs. RHE), *FE* is the selectivity of NO_3_^−^‐to‐NH_3_, *P_light_* is the input power of the system (100 mW cm^−2^), *A* was the area of the solar cell (3.8 cm^2^).

### 5.9 Plant Growth Experiment

Equal quantities of lettuce, rapeseed, and cabbage seeds were sown in rows within hydroponic containers. The control group received a nutrient solution supplemented with KNO_3_, while the experimental group was irrigated daily with the electrolyte generated via STA electrolysis reduction. Carbon dioxide gas was introduced to maintain the solution pH at approximately 7. After a 45‐day growth period, plant biomass and root‐shoot parameters were measured for comparative analysis.

### 5.10 CO_2_ Adsorption Capacity Measurement

At room temperature and atmospheric pressure, 15 mL of NITRR electrolytes or other test solutions were used as absorbents. CO_2_ was introduced at a constant flow rate of 10 mL·min^−1^ for 40 min. Selected solutions were evaluated in triplicate to determine absorption capacity. CO_2_ uptake was quantified via titration with a standardized HCl solution (0.6098 M). First, a drop of phenolphthalein indicator was added to the sample after CO_2_ absorption. HCl was then titrated slowly until the pink color faded, ensuring complete conversion of CO_3_2^−^ to HCO_3_^−^ and neutralizing any residual OH^−^ or NH_3_. Subsequently, nine drops of a mixed indicator (bromocresol green and methyl red) were introduced. Titration with HCl continued until the solution turned from green to pink. The resulting mixture was boiled, cooled, and a final small amount of HCl was added to restore the pink endpoint. The total volume of HCl consumed in the last two titration steps was used to calculate the amount of captured CO_2_, with the molar equivalent of HCl corresponding directly to the moles of CO_2_ absorbed.

### 5.11 Dry Weight Determination

Each sample was first rinsed with deionized water, followed by gently blotting surface moisture with absorbent paper. The samples were immediately sterilized in a 105°C oven for 120 min. Subsequently, the samples were cooled to 70–80°C and baked until reaching a constant weight, after which the dry biomass was measured.

## Author Contributions

R.Y. conceived and designed the research. R.Y. and A.K.Y.J. supervised the research. W.G. carried out most of the experiments. X.H. and A.J. provide perovskite cells and corresponding data for testing. Y.M., Y.S., Z.L., Q. Z. and Y. X. assisted with other experiments. S.X. performed the x‐ray absorption spectroscopy experiment and J.W. assisted data analysis. R.Y. and W.G. analyzed the data and wrote the manuscript with input from the other authors.

## Conflicts of Interest

The authors declare no conflicts of interest.

## Supporting information




**Supporting File 1**: anie72479‐sup‐0001‐SuppMat.Pdf.

## Data Availability

The data that support the findings of this study are available from the corresponding author upon reasonable request.
