# Supplementary material for: Solar‐Driven Ammonia Synthesis From Nitrate Reduction Paired With CO2 Capture for Sustainable Agriculture via a Robust CuPd Heterojunction
Source: Angew Chem Int Ed Engl. 2026 May 2;65(25):e9278631. doi: 10.1002/anie.9278631 (PMC13266953; doi:10.1002/anie.9278631)
Supplement: Supplementary file 1 — Supporting File 1: anie72479‐sup‐0001‐SuppMat.Pdf. [file ANIE-65-e9278631-s001.pdf]

Solar-Driven Ammonia Synthesis from Nitrate Reduction Paired with CO_2 Capture for Sustainable Agriculture via a Robust CuPd Heterojunction

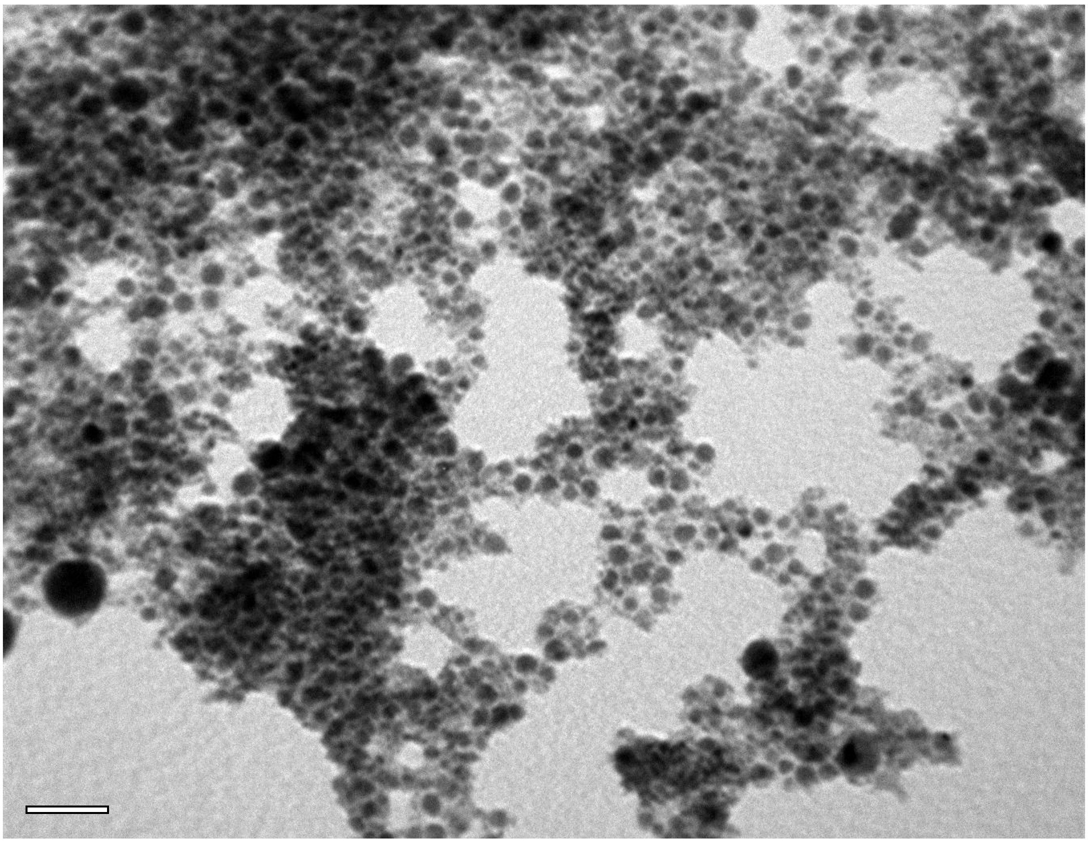

Figure S1. Structural characterization of L-CuPd. TEM image of L-CuPd.

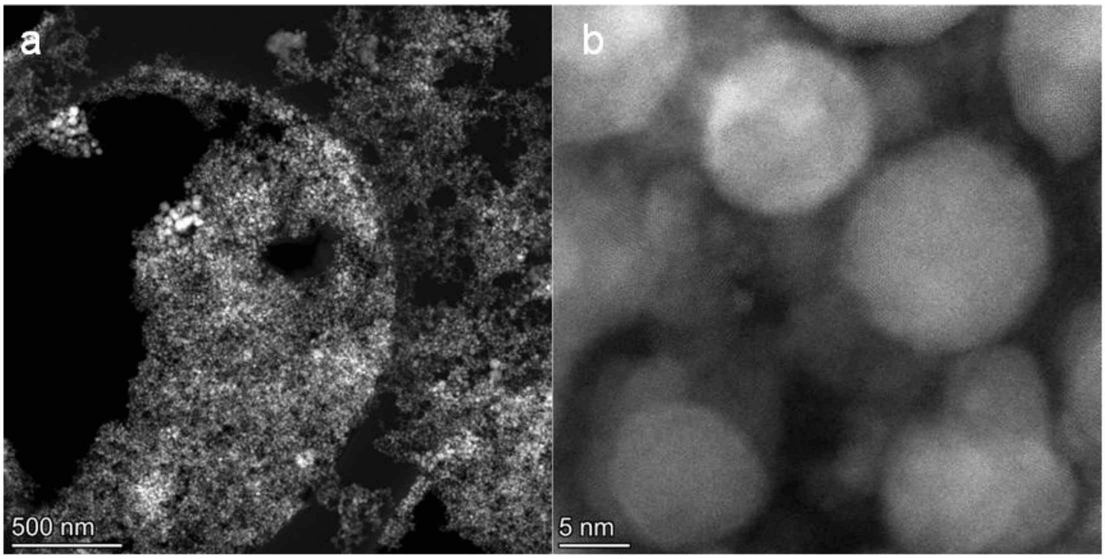

Figure S2. Structural characterization of L-CuPd. STEM image of L-CuPd.

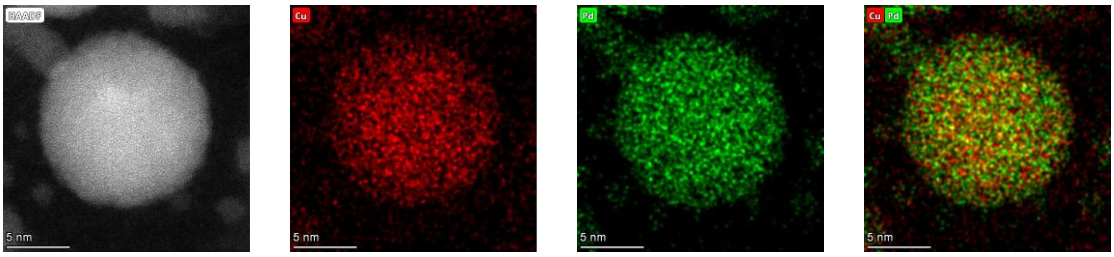

2023-03-17 09:48:53 Analysis of spectrum: Spectra from Area #1

Z	Element	Family	Atomic Fraction (%)	Atomic Error (%)	Mass Fraction (%)	Mass Error (%)	Fit error (%)
29	Cu	K	34.92	5.67	24.26	2.66	0.23
46	Pd	L	65.08	12.35	75.74	11.15	0.16

Figure S3. Composition characterization of L-CuPd. EDS mapping and spectra of ratio of Cu and Pd.

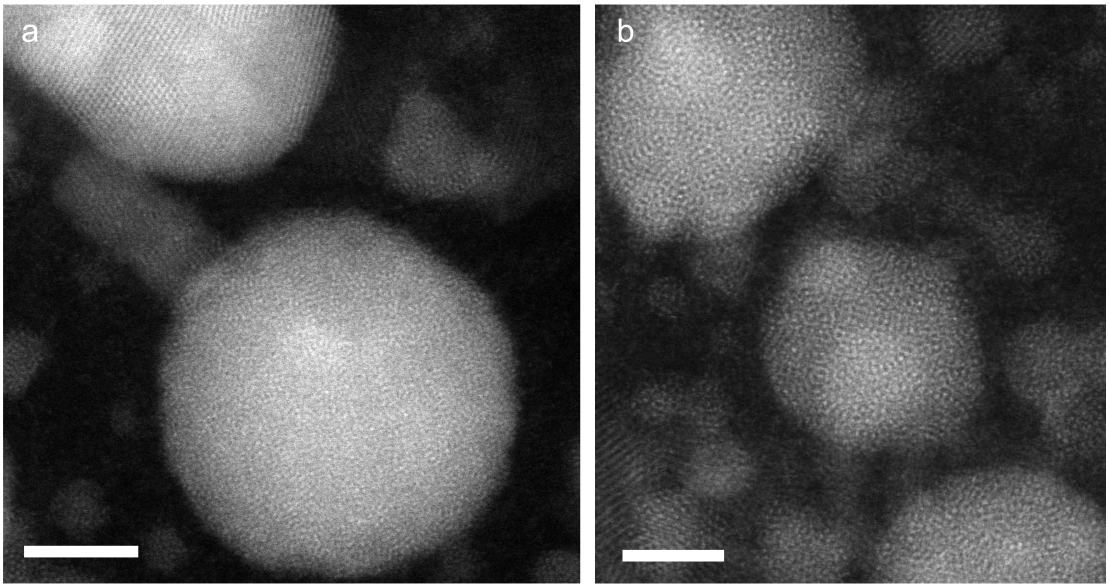

Figure S4. High-resolution HAADF-STEM image of L-CuPd. Scar bar: 5nm in (a) and (b).

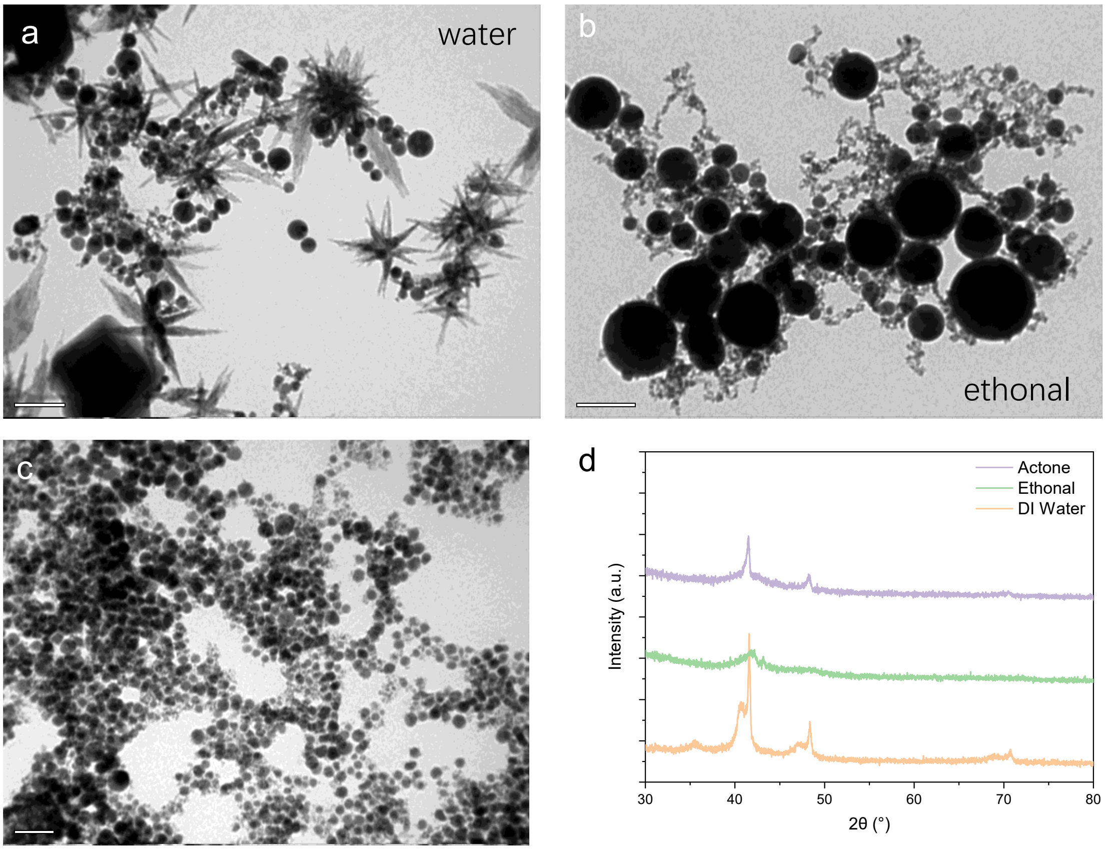

Figure S5. Structural characterization of the laser in different solvents. TEM of L-CuPd synthesis in (a) DI water, (b) Ethanol, and (c) Acetone. XRD of L-CuPd in different solvents. L-CuPd with amorphous and crystalline Interfaces is synthesis in actone.

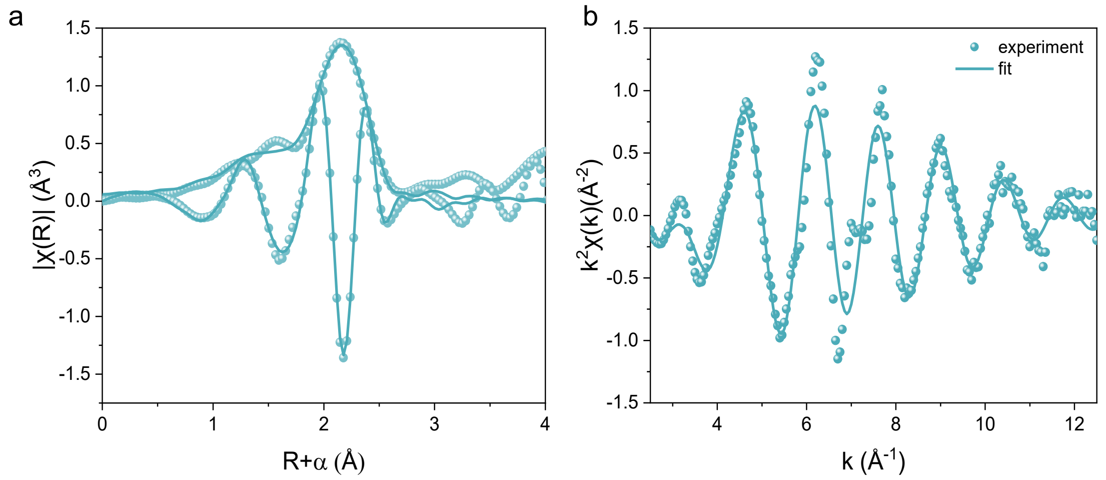

Figure S6. Fitting results of Cu K-edge EXAFS spectra of L-CuPd. Fourier transform of Cu K-edge EXAFS fitting results of (a) R-space, (b) k-space.

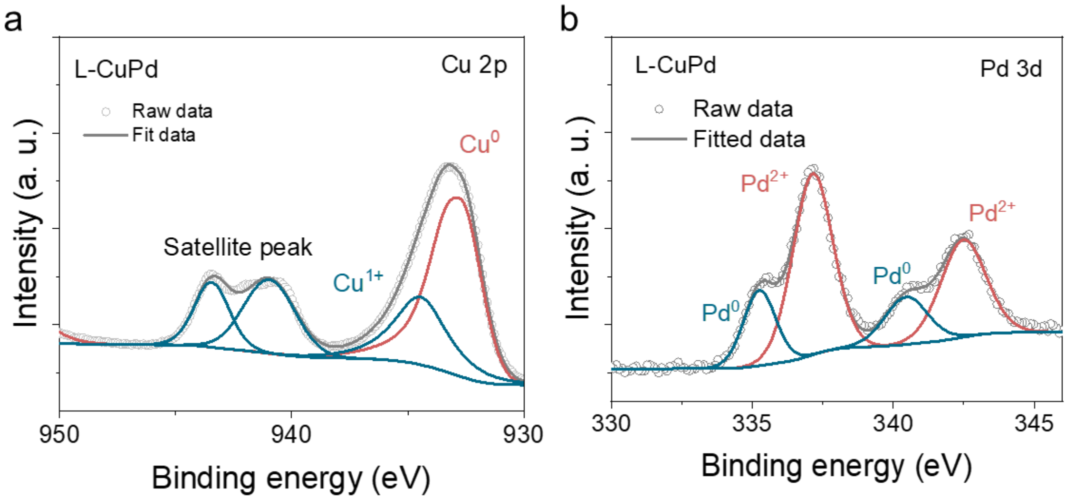

Figure S7. Structural characterization of L-CuPd. Full spectrum of (a) Cu 2p XPS spectra and (b) Pd 3d XPS spectra.

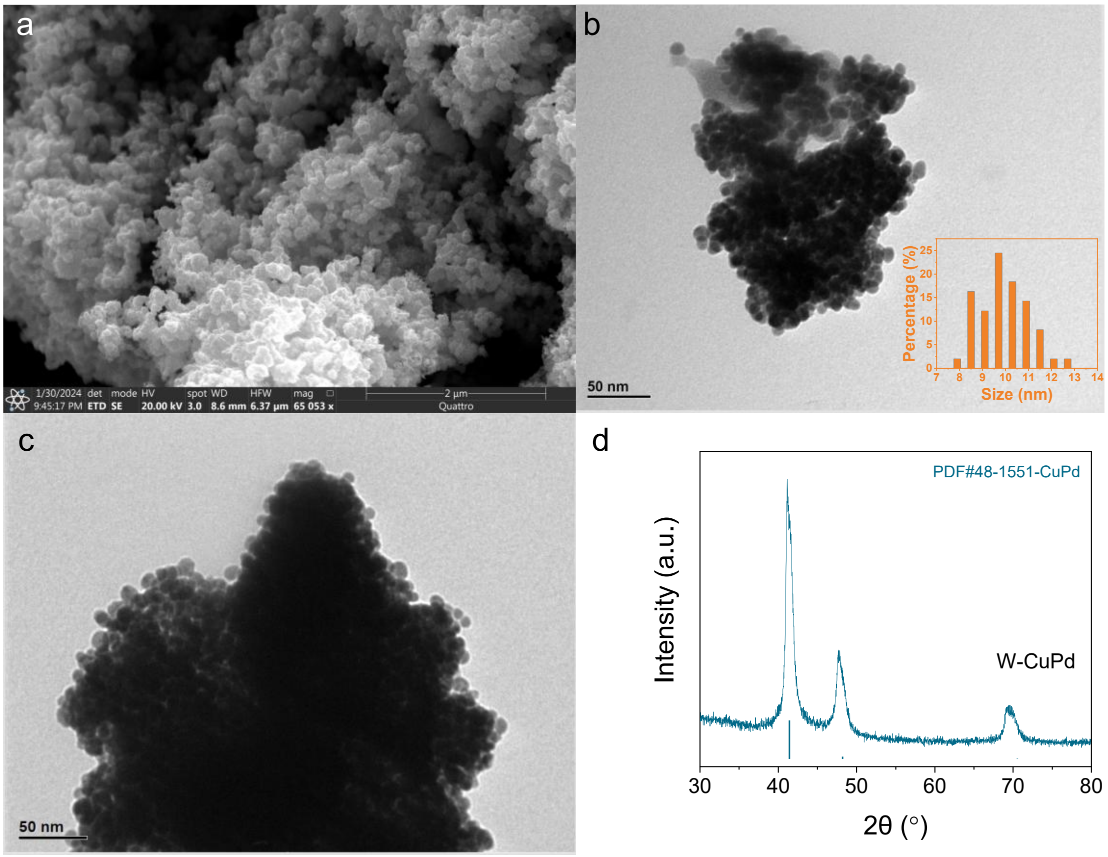

Figure S8. Structural characterization of W-CuPd. (a) SEM image, (b,c) TEM images, and (d) XRD pattern of W-CuPd.

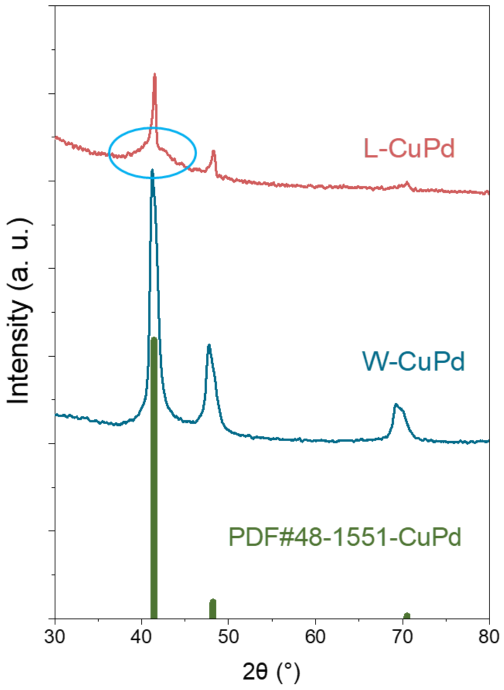

Figure S9. XRD pattern comparison of L-CuPd and W-CuPd. The blue circles represent amorphous diffraction peaks.

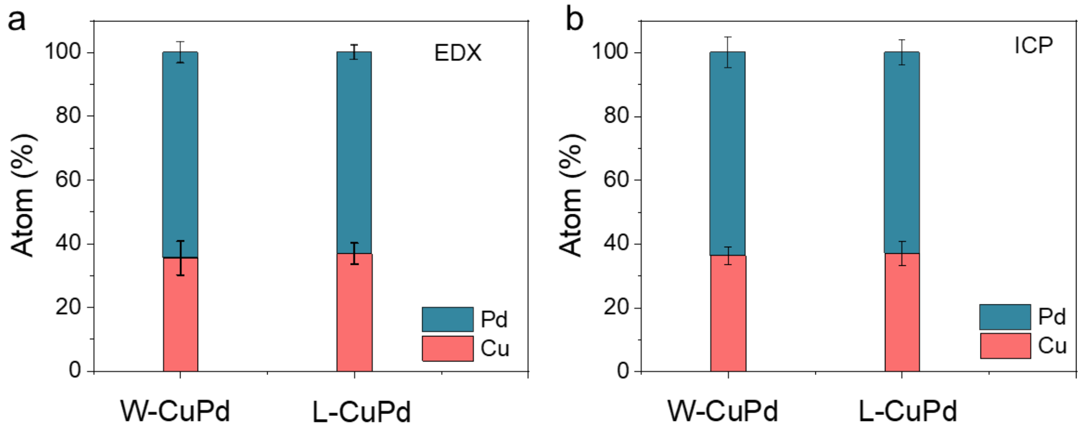

Figure S10. EDX and ICP results of W-CuPd and L-CuPd comparison.

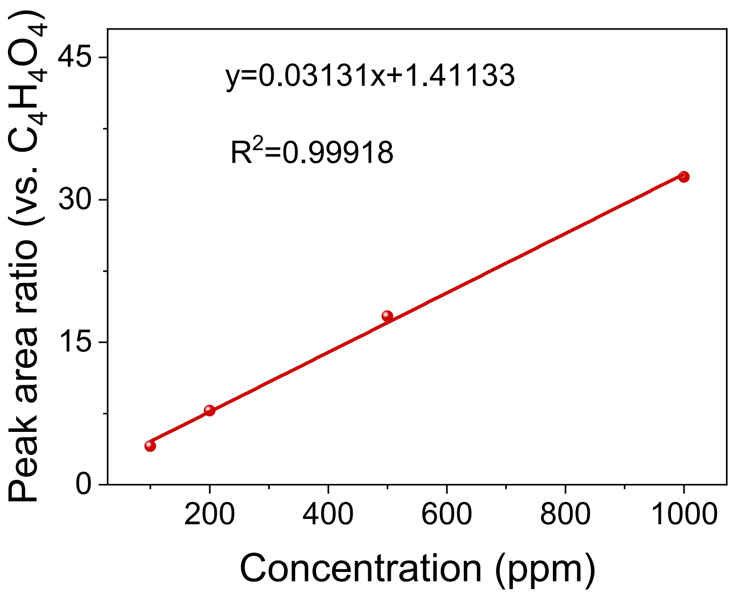

Figure S11. **Calibration curves.** ^{1}H NMR calibration curve of NH_3 using different ammonium chloride concentration solutions of known concentration as standards.

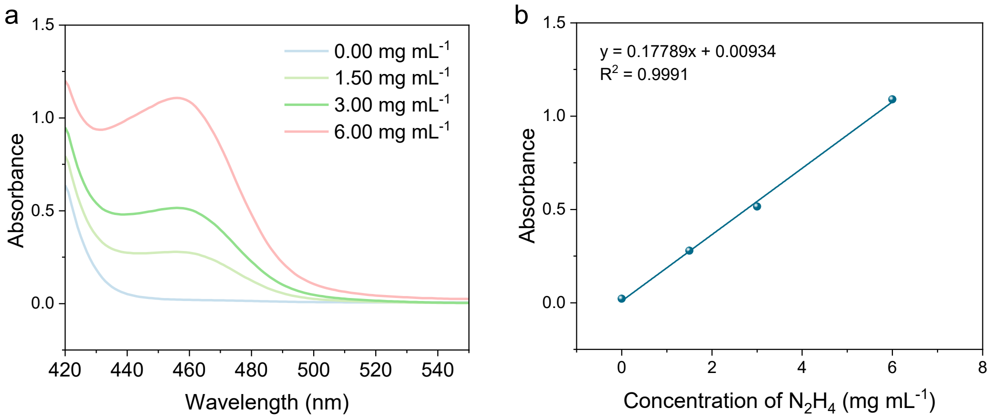

Figure S12 The standard curve of N_2H_4 obtained by the Watt and Chrisp method. (a) UV-vis absorption spectra of different N_2H_4 concentrations. (b) Linear fitting result of the standard curves.

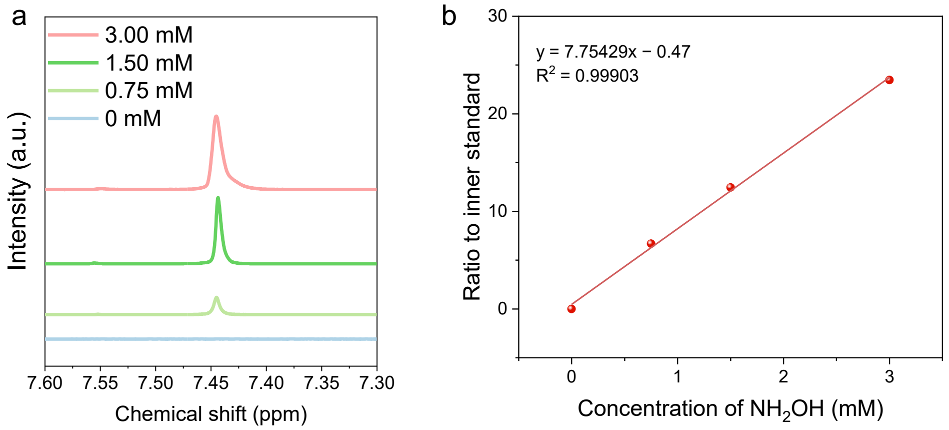

Figure S13. The standard curve of NH_2OH obtained by the 1H NMR method. (a) 1H NMR spectra of different NH_2OH concentrations. (b) Linear fitting result of the standard curves.

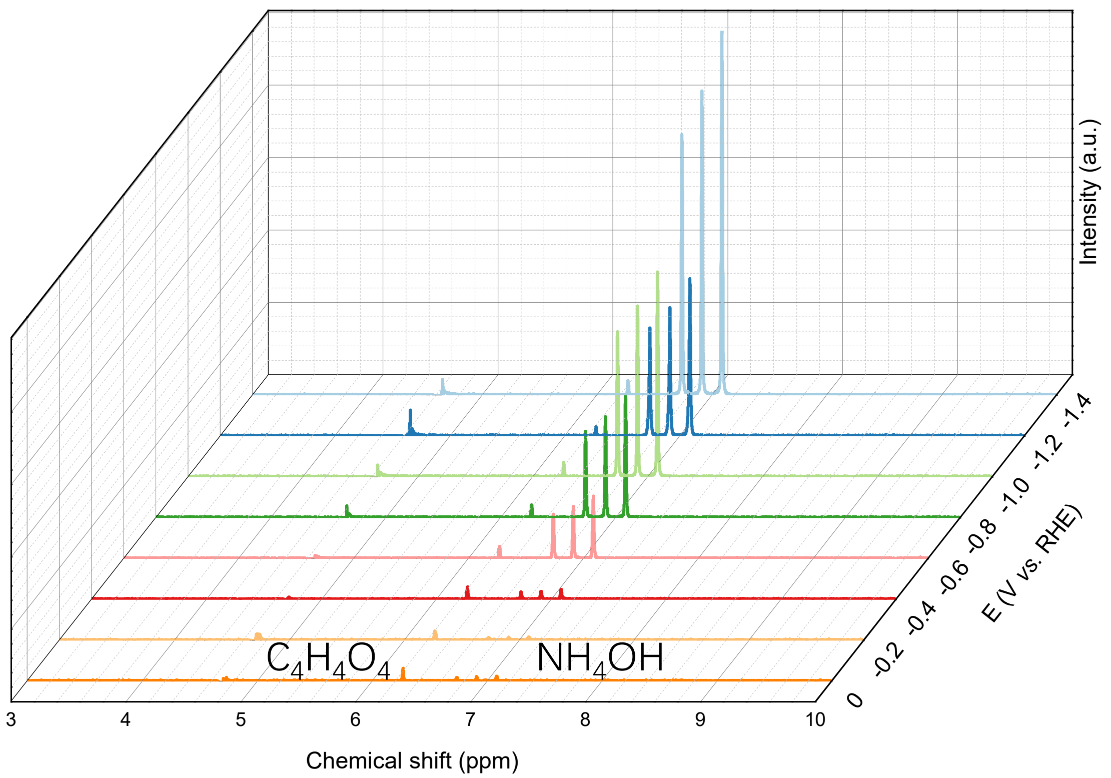

Figure S14. Product analysis. Representative 1H-NMR spectrum of liquid products collected from the cathode side.

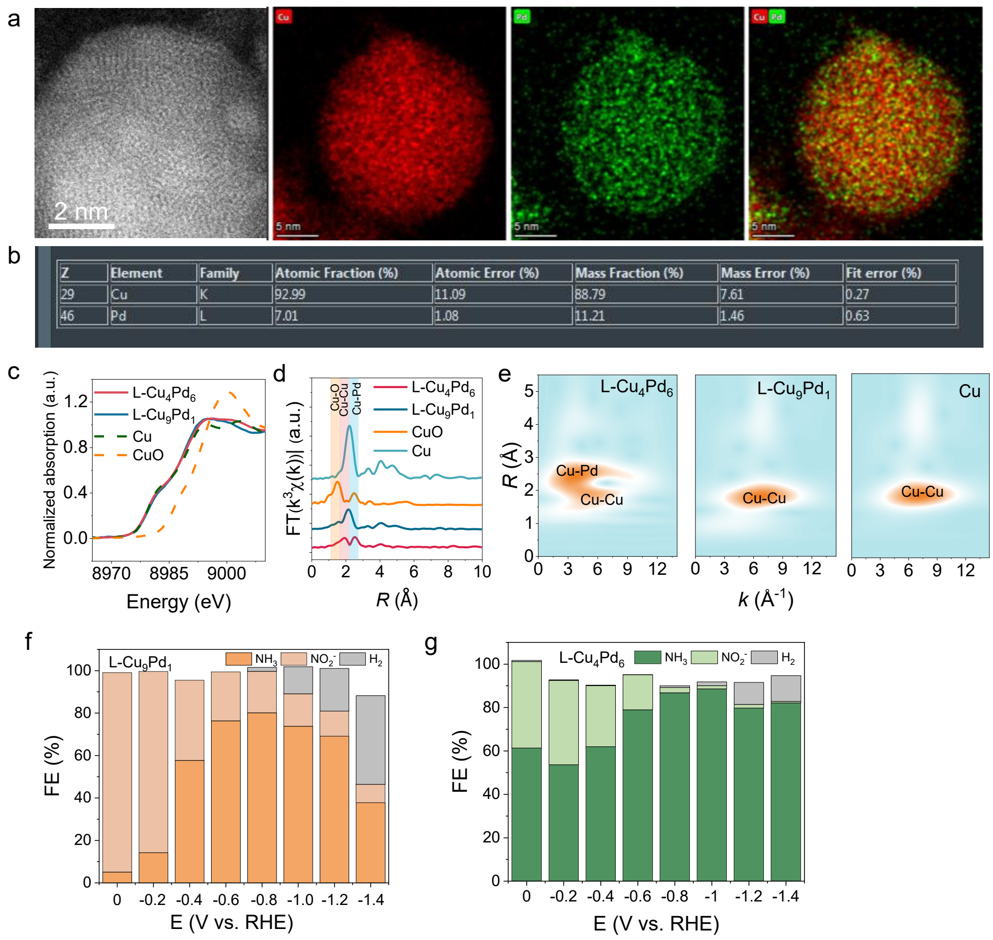

Z	Element	Family	Atomic Fraction (%)	Atomic Error (%)	Mass Fraction (%)	Mass Error (%)	Fit error (%)
29	Cu	K	92.99	11.09	88.79	7.61	0.27
46	Pd	L	7.01	1.08	11.21	1.46	0.63

Figure S15. Composition characterization of L-Cu_9Pd_1. (a) HAADF-STEM image, EDS mapping and (b) spectra of ratio of Cu and Pd. (c) Normalized XANES, (d) Fourier transformed EXAFS and (e) WT–EXAFS Cu K-edge spectra of L-Cu_4Pd_6, L-Cu_9Pd_1, Cu and CuO. (f) Faraday efficiency of L-Cu_9Pd_1 and (g) L-Cu_4Pd_6 in different potentials. To avoid confusion, L-Cu_4Pd_6 and L-Cu_9Pd_1 are specifically labeled in the table below for distinction. Elsewhere, unless otherwise specified, L-CuPd refers to L-Cu_4Pd_6.

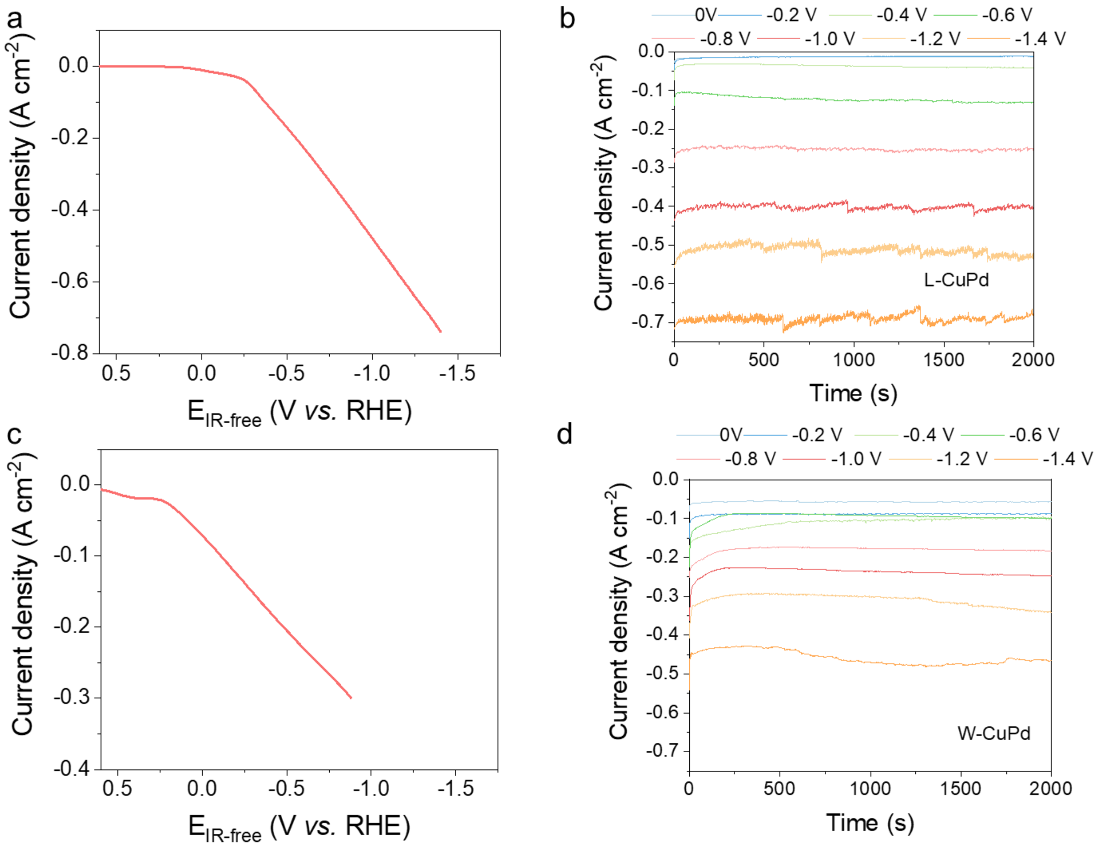

Figure S16. Electrochemical performances. (a and c) Chronoamperometry and (b and d) chronoamperometry of L-CuPd and W-CuPd at different potentials in 1 M KOH + 1 M KNO_3.

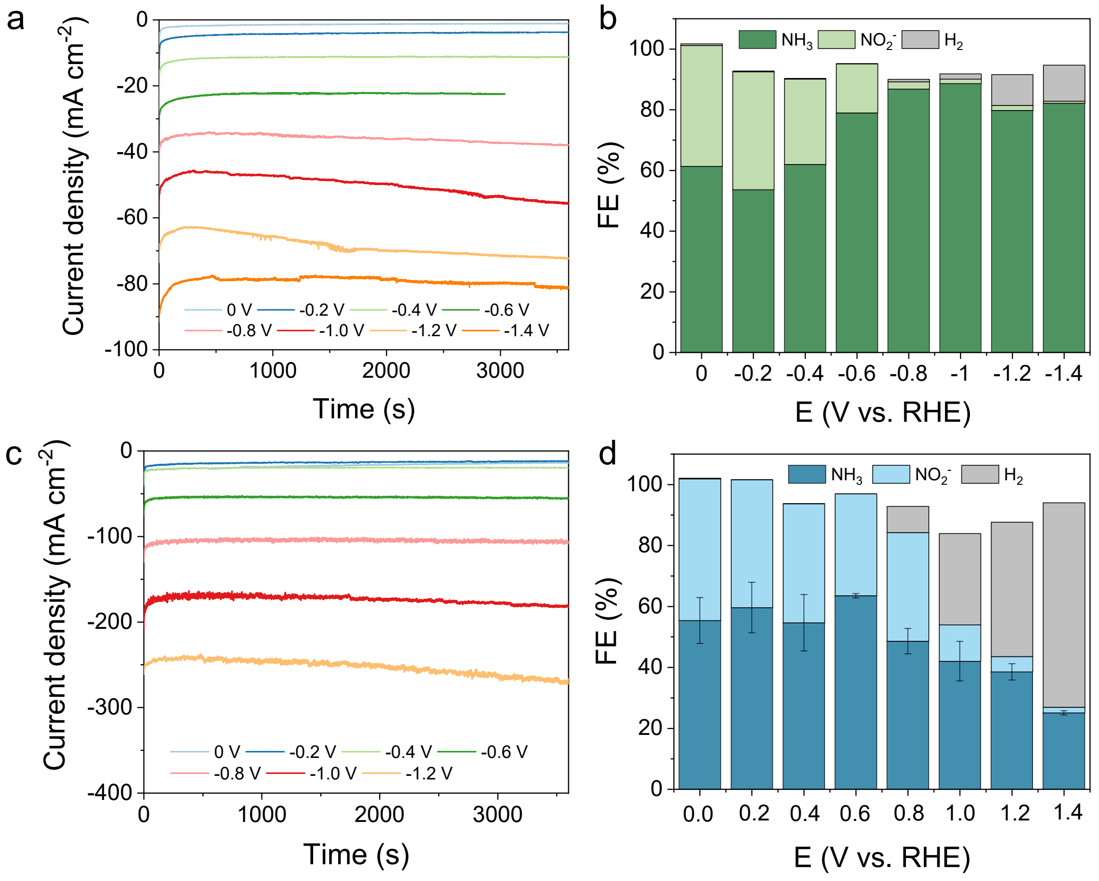

Figure S17. Electrochemical performances. (a and c) Chronoamperometry and (b and d) FE of L-CuPd in 1 M KOH + 0.05 M KNO_3, and 1 M KOH + 0.1 M KNO_3 .

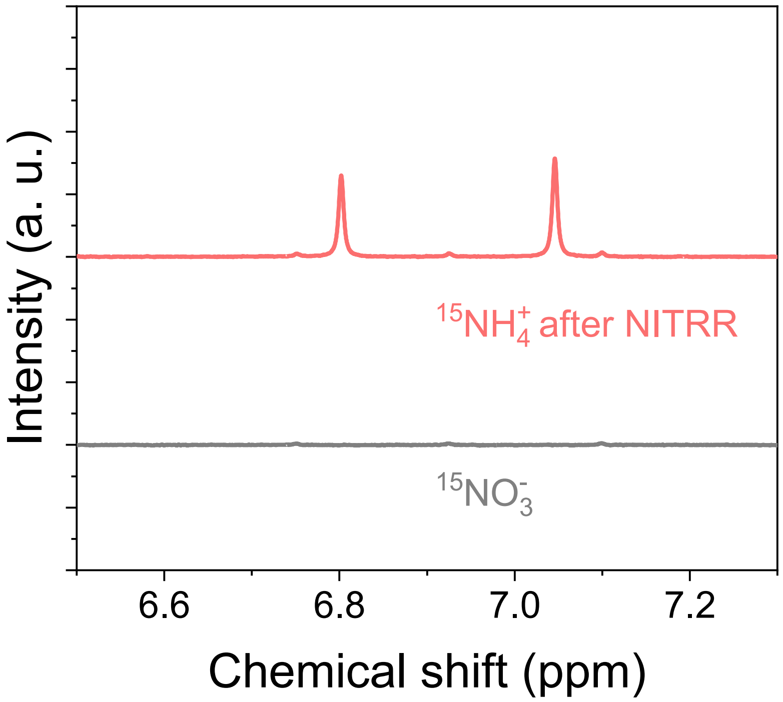

Figure S18. Product analysis. Representative 1H-NMR spectrum of NMR spectra after NITRR using $^{15}NO_3^-$ and $^{15}NO_3^-$ electrolytes.

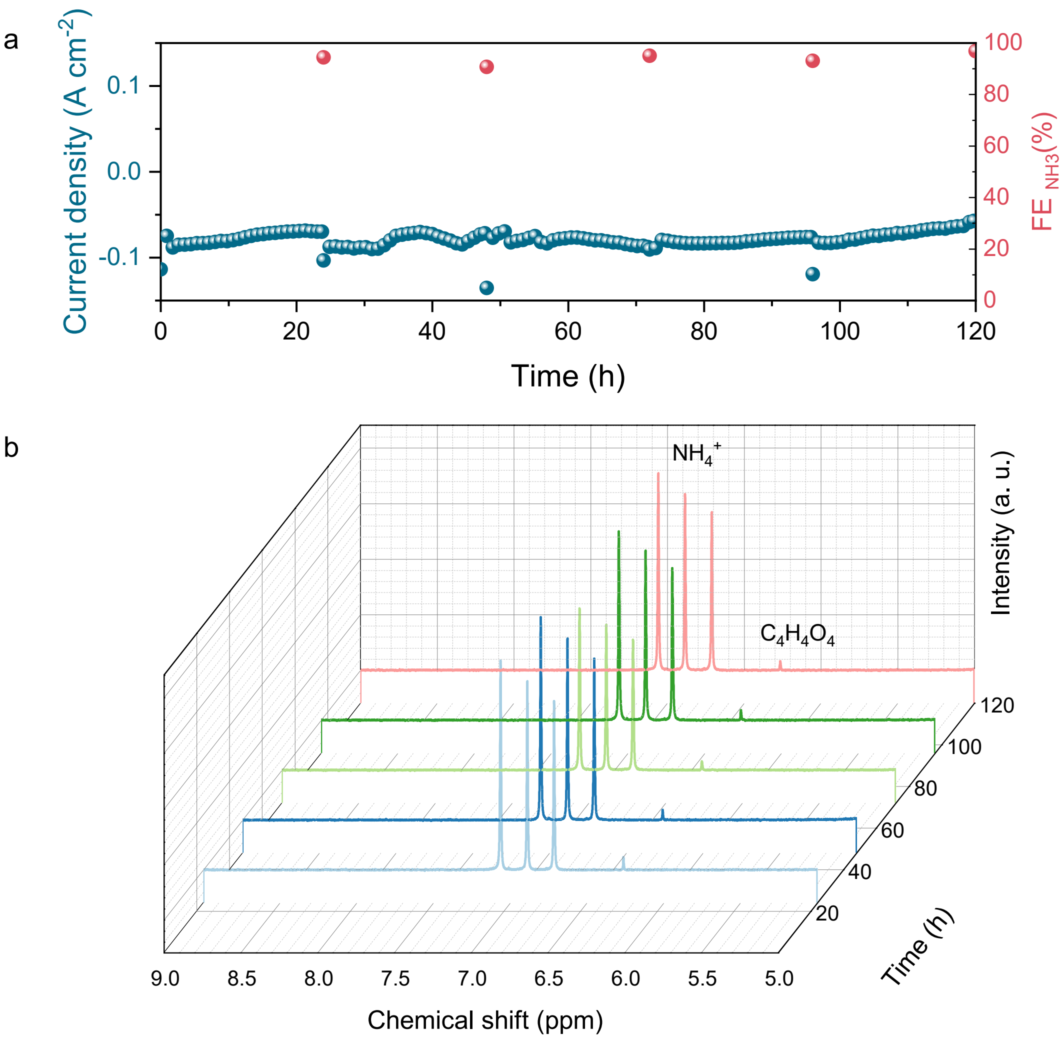

Figure S19. (a) j Stability test of solar-driven system under 1 sun illumination at 90 mA cm^{-2} and (b) the corresponding ammonia production in NMR.

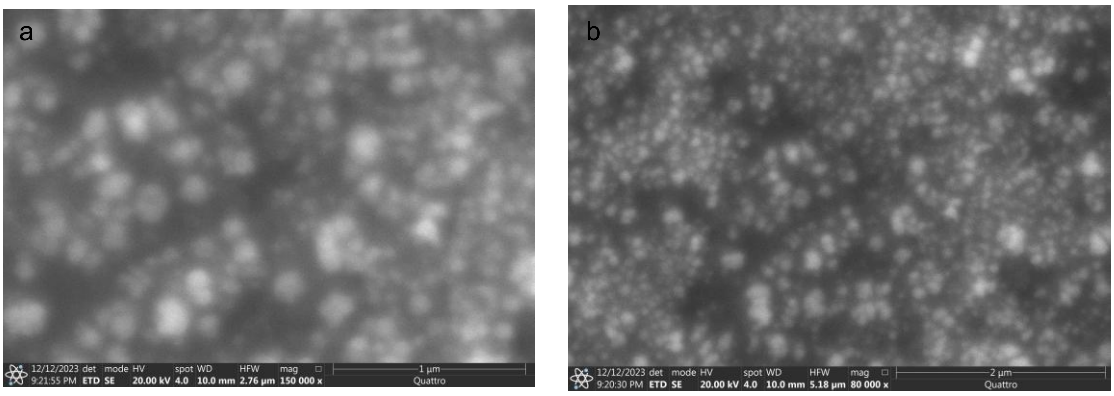

Figure S20. Composition characterization of L-CuPd. SEM images after 100 h of stability.

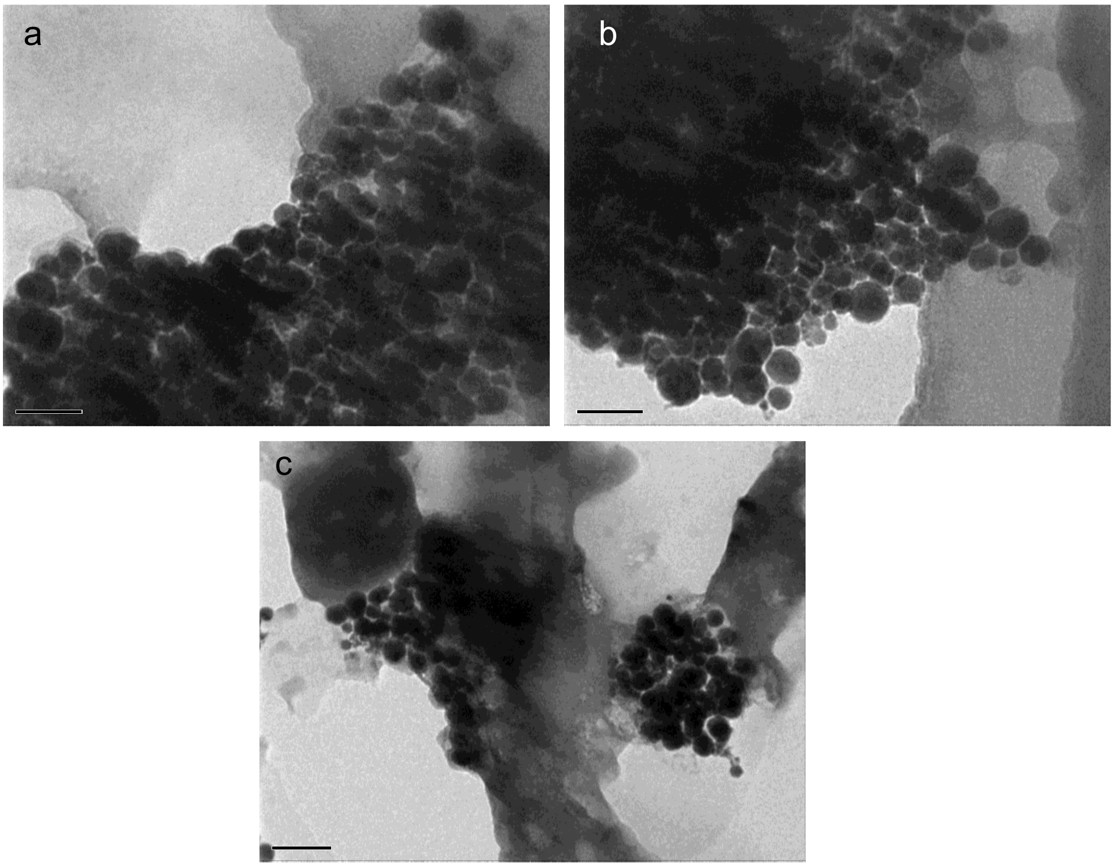

Figure S21. Composition characterization of L-CuPd. TEM images after 100 h of stability.

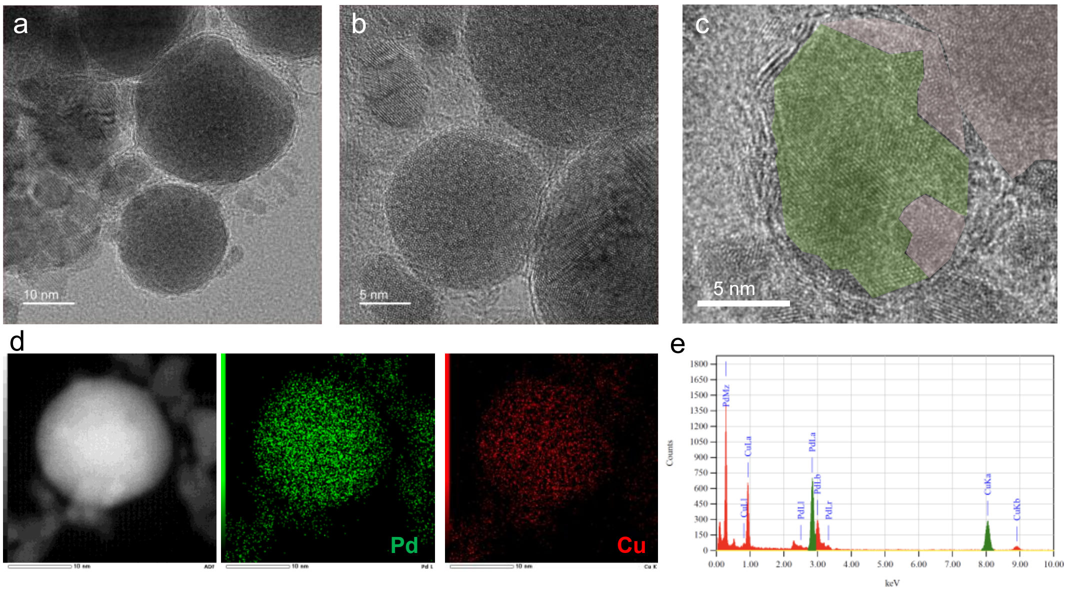

Figure S22. (a-c) HRTEM images of L-CuPd characterization after stability. Pink area: amorphous; Green area: crystalline. (d) EDX mapping and corresponding EDX of Cu and Pd after stability.

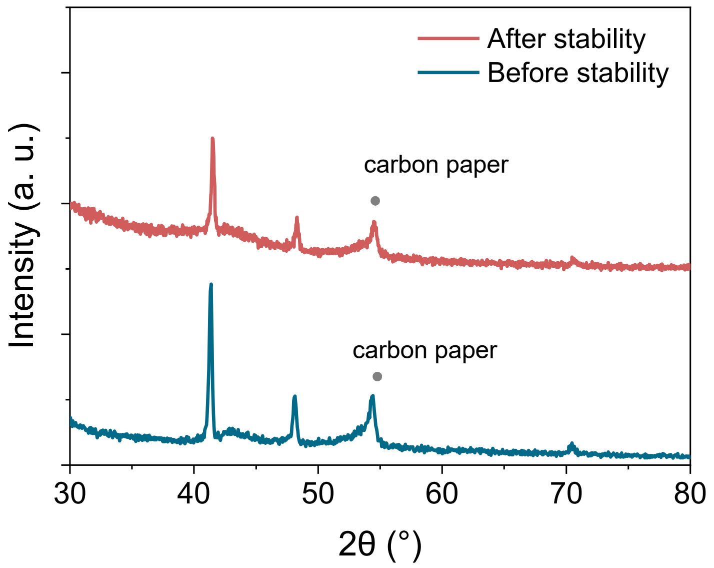

Figure S23. XRD pattern of L-CuPd before and after stability.

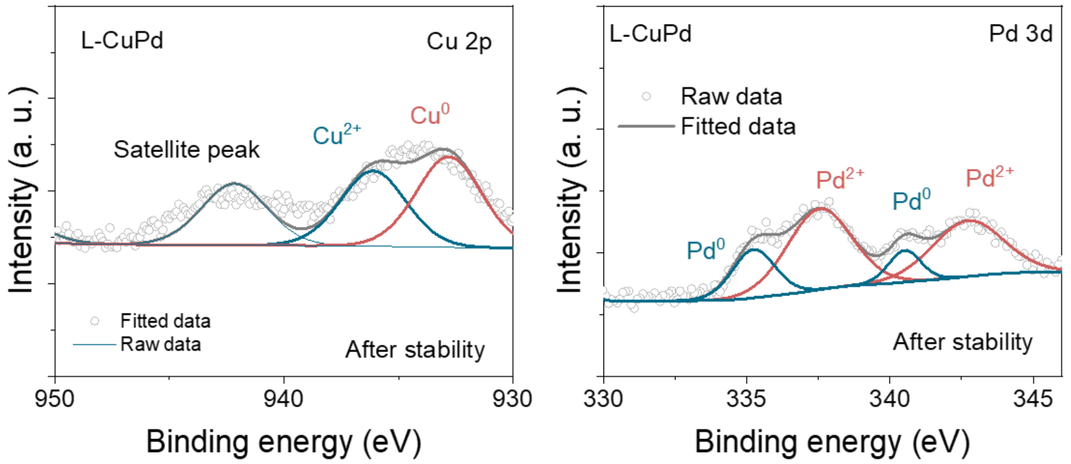

Figure S24. Structural characterization of after stabilization. Full-spectrum of (a) Cu 2p XPS spectra and (b) Pd 3d XPS spectra.

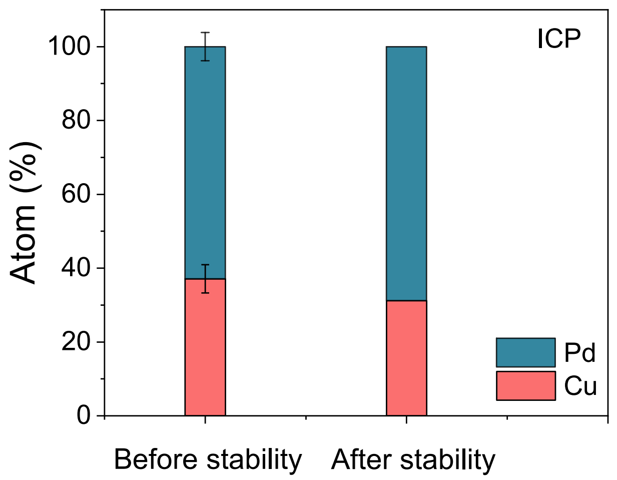

Figure S25. ICP comparison of the ratio Cu and Pd before and after stability.

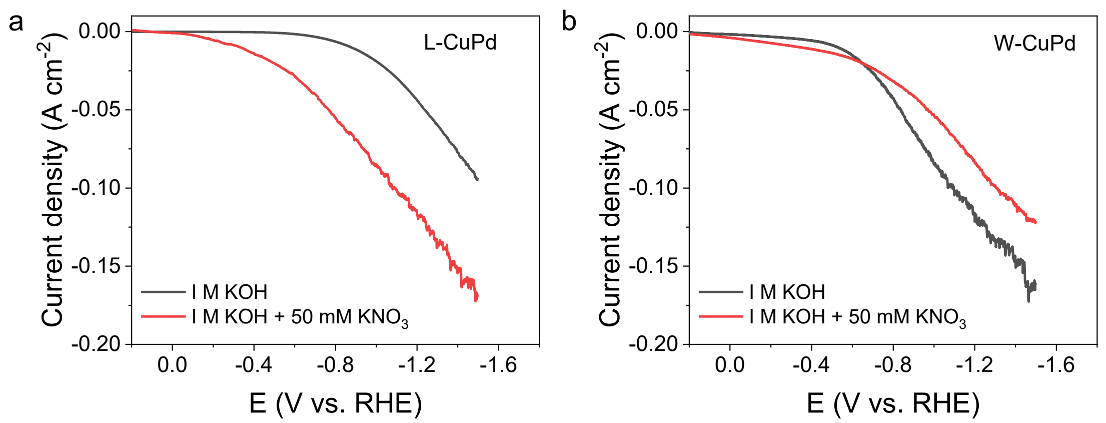

Figure S26. Electrochemical performances. The LSV of NTIRR and HER in 1 M KOH + 0.05 M KNO_3 + 1 M KOH of (a) L-CuPd and (b) W-CuPd.

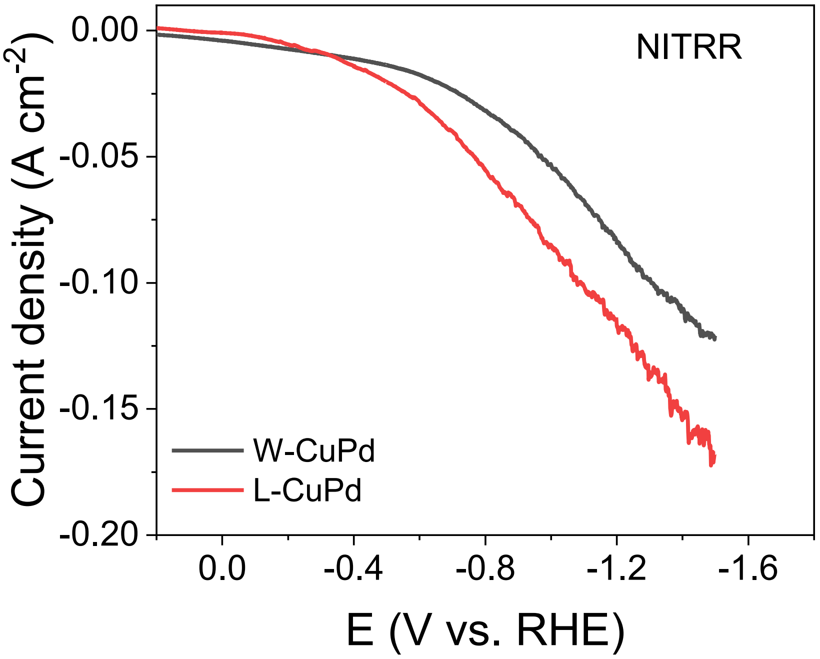

Figure S27. Electrochemical performances. Comparison of LSV during NITRR in 1 M KOH + 0.05 M KNO_3 of L-CuPd and W-CuPd.

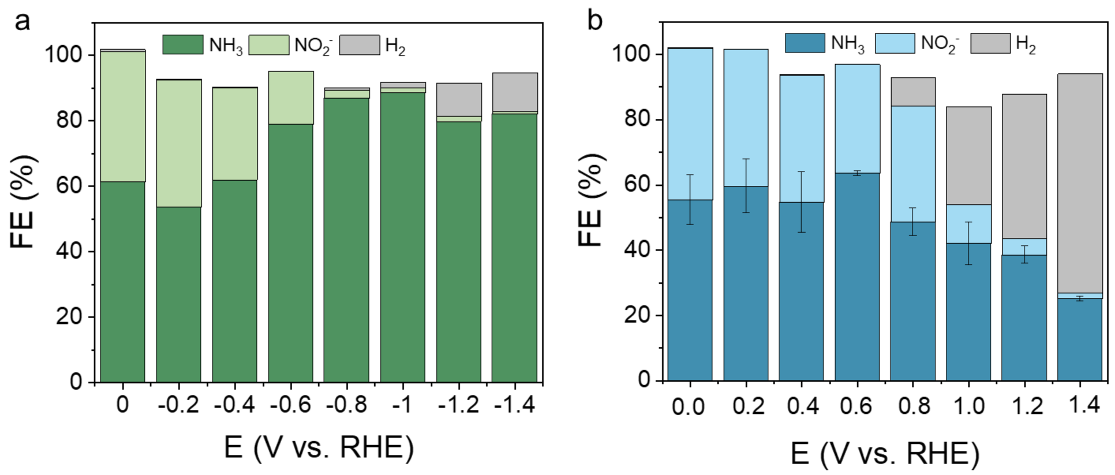

Figure S28. Electrochemical performances. The FE of all products during NITRR in 1 M KOH + 0.05 M KNO_3 of (a) L-CuPd and (b) W-CuPd.

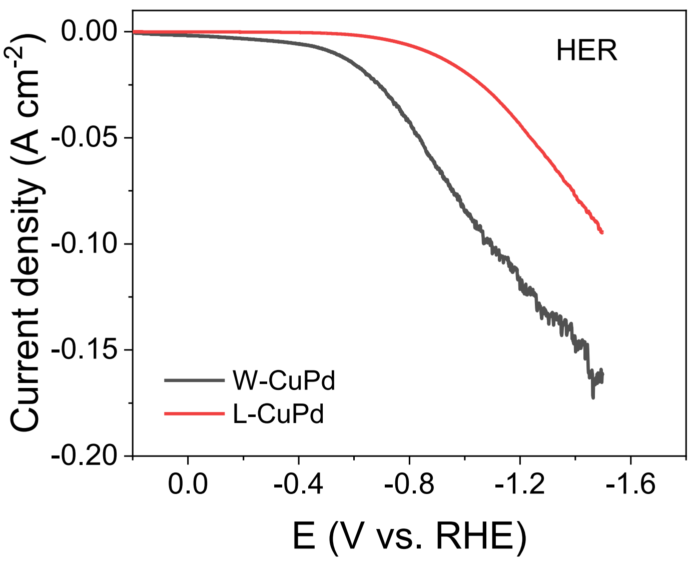

Figure S29. Electrochemical performances. Comparison of HER in 1 M KOH of L-CuPd and W-CuPd.

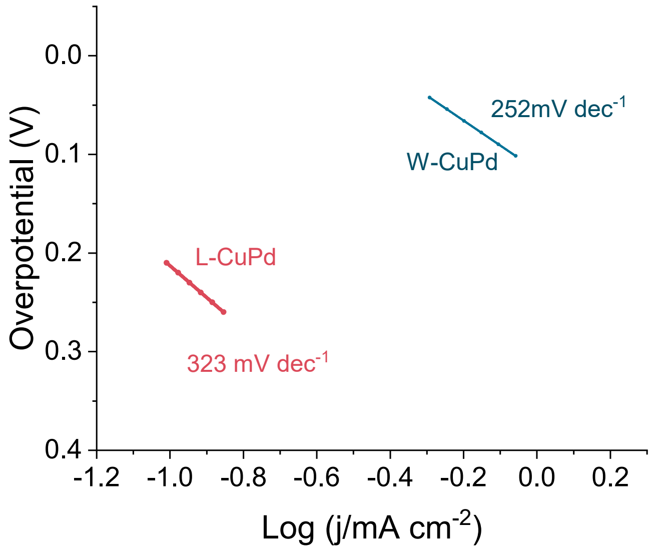

Figure S30. Tafel plot of the L-CuPd and W-CuPd electrodes derived from Figure S29.

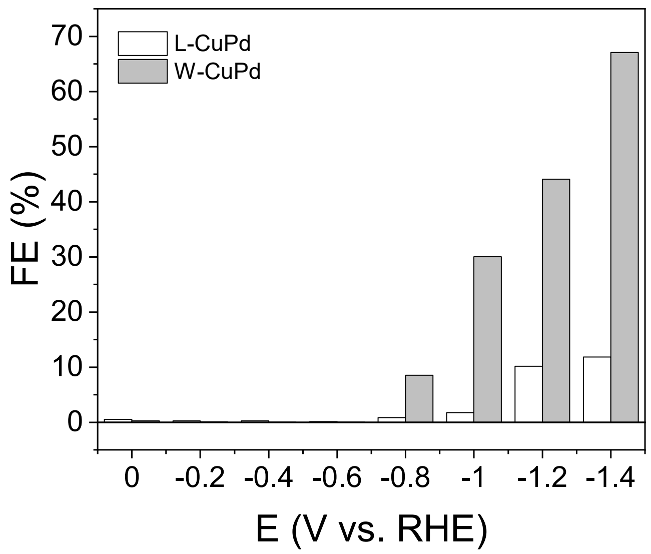

Figure S31. Electrochemical performances. Comparison of FE of H_2 in 1 M KOH + 0.05 M KNO_3 of CuPd and CuPd.

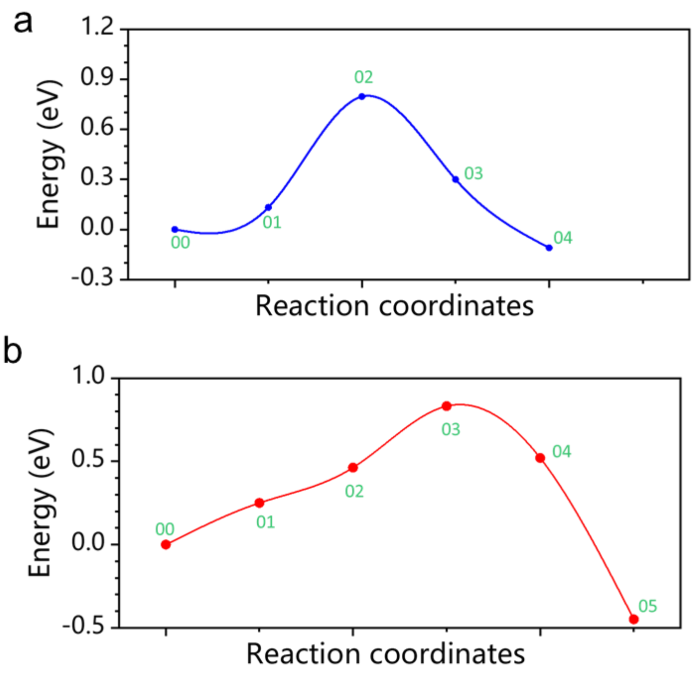

Figure S32. DFT simulation energy profiles of H_2O on (a) W-CuPd (111) slab, with an activation energy barrier of +0.80 eV and (b) L-CuPd(111) interface, with an activation energy barrier of +0.83 eV.

DFT calculations reveal that the activation energy barrier for H_2O dissociation on W-CuPd(111) is 0.80 eV, which is 0.03 eV lower than that on L-CuPd(111) (0.83 eV). This slight but distinct difference is consistent with the experimental HER performance, where W-CuPd exhibits superior activity, including a more positive onset potential, lower overpotential, and higher current density compared to L-CuPd.

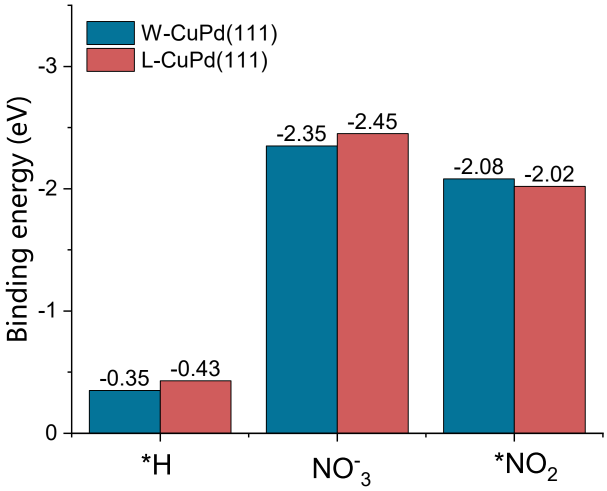

Figure S33. DFT-calculated free energies of key intermediates (NO_3^-, $*NO_2$, and *H) on W-CuPd(111) and L-CuPd(111) surfaces for nitrate electroreduction.

From these results, the following observations can be made: (1) NO_3^- adsorption is stronger on L-CuPd(111) (−2.45 eV) than on W-CuPd(111) (−2.35 eV), with a difference of 0.10 eV. This stronger adsorption facilitates the initial activation of nitrate ions, which is beneficial for the overall reduction process. (2) $*NO_2$ intermediate binds more strongly on W-CuPd(111) (−2.08 eV) compared to L-CuPd(111) (−2.02 eV). The slightly weaker $*NO_2$ adsorption on L-CuPd(111) may lower the energy barrier for subsequent N–O bond cleavage, promoting faster conversion to NH_3.

(3) *H adsorption is stronger on L-CuPd(111) (−0.43 eV) than on W-CuPd(111) (−0.35 eV), in this case it may also facilitate hydrogenation steps essential for nitrate reduction, consistent with the superior performance of L-CuPd.

These DFT results indicate that L-CuPd(111) exhibits stronger NO_3^- adsorption and moderate $*NO_2$ binding, which together contribute to its superior nitrate reduction performance compared to W-CuPd(111). The stronger *H adsorption on L-CuPd may also assist in the hydrogenation steps required for complete nitrate conversion.

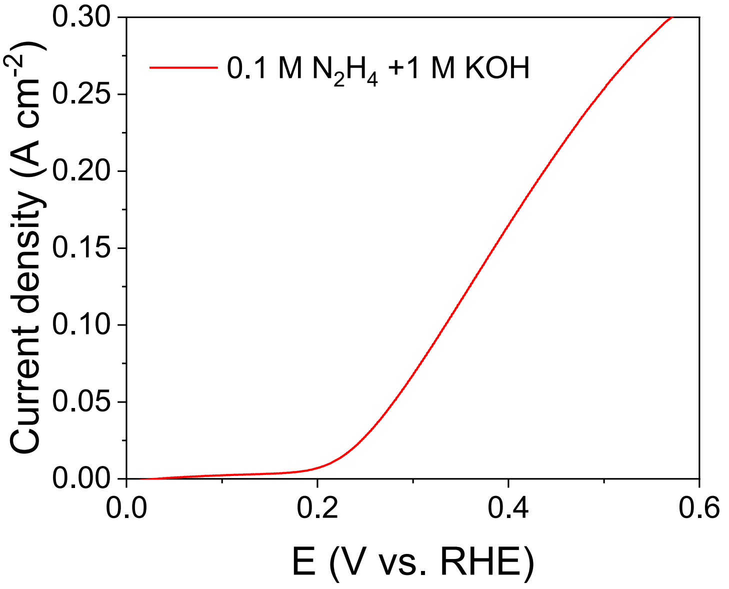

Figure S34. Electrochemical performances. LSV of hydrazine oxidation reaction (HzOR) of in 1 M KOH + 0.1 M N_2H_4OH.

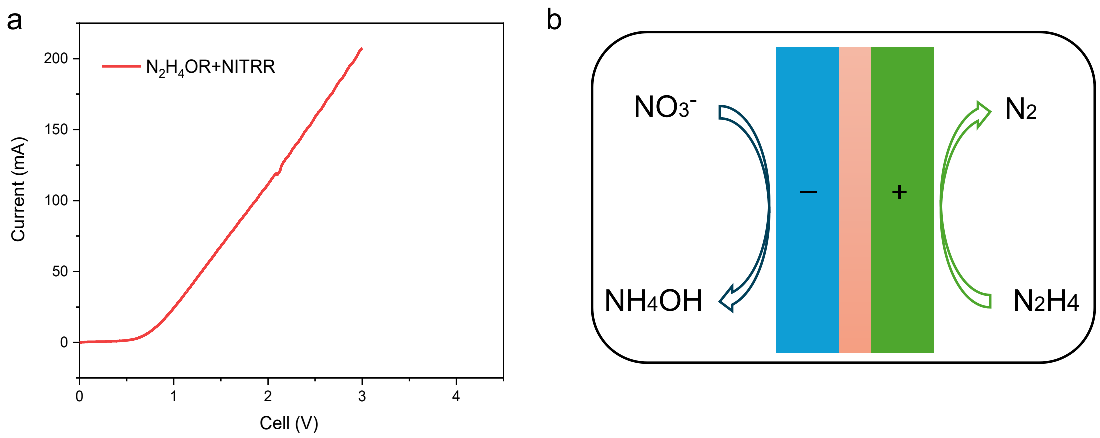

Figure S35. Nitrate and hydrazine-containing wastewater. (a) LSV and (b)Schematic diagram of the reaction process.

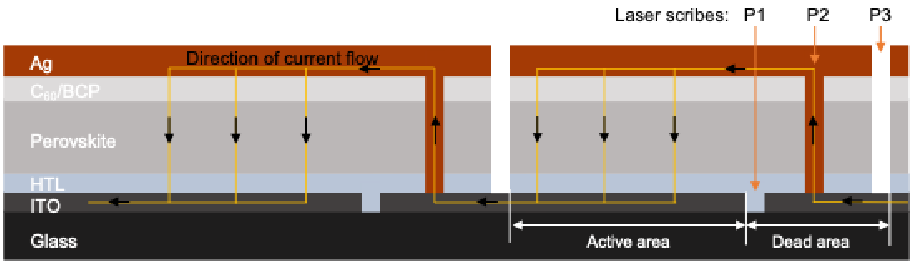

Figure S36. Schematic of the perovskite solar module, with the active area of each subcell set to approximately 3.8 cm^2. series-connected two-junction perovskite solar minimodules fabricated via laser scribing to enable electrical interconnection between adjacent subcells.

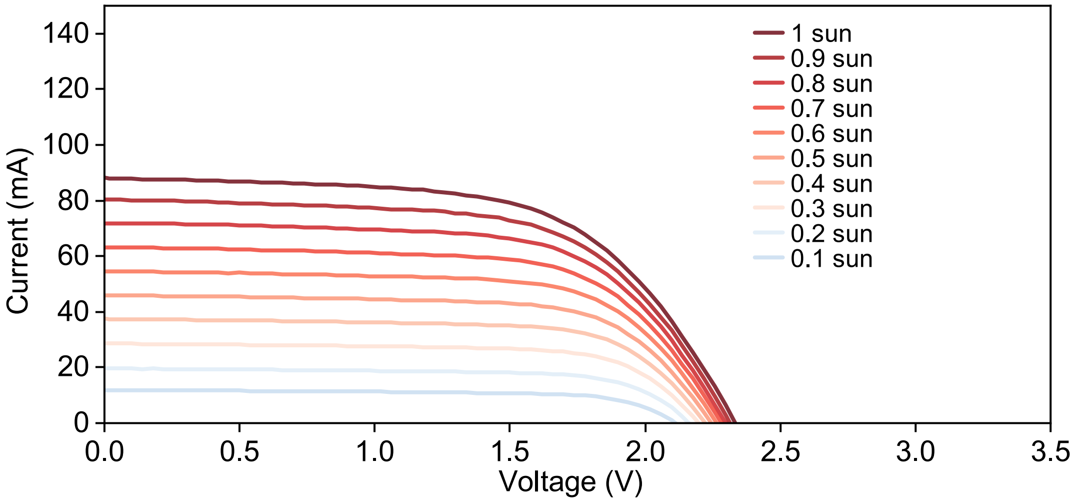

Figure S37. Light-dependent device performance.

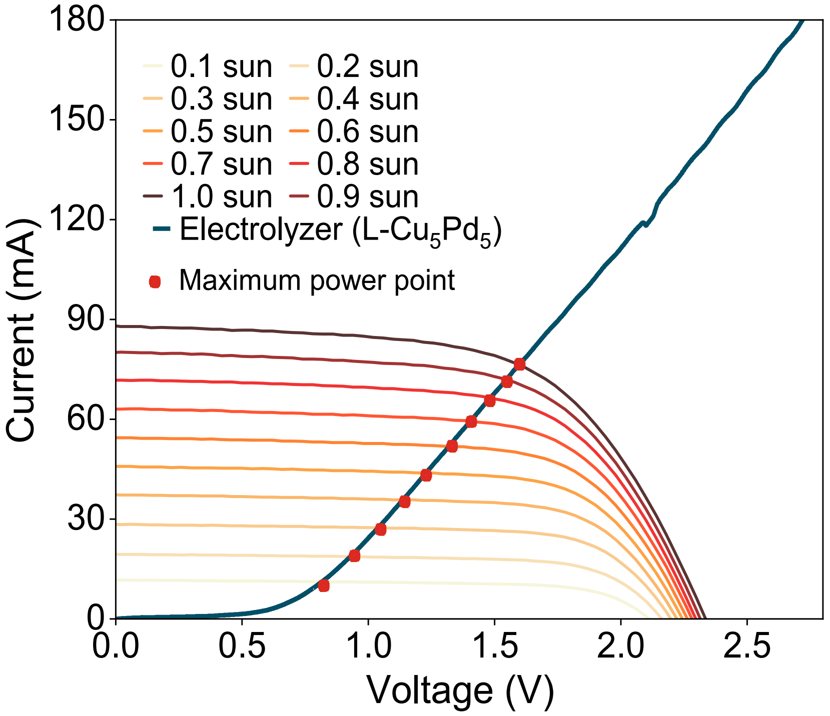

Figure S38. Intersections of JV characteristics of perovskite solar cells with the load curve of an electrochemical cell of L-CuPd and the optimal operating point at different sun.

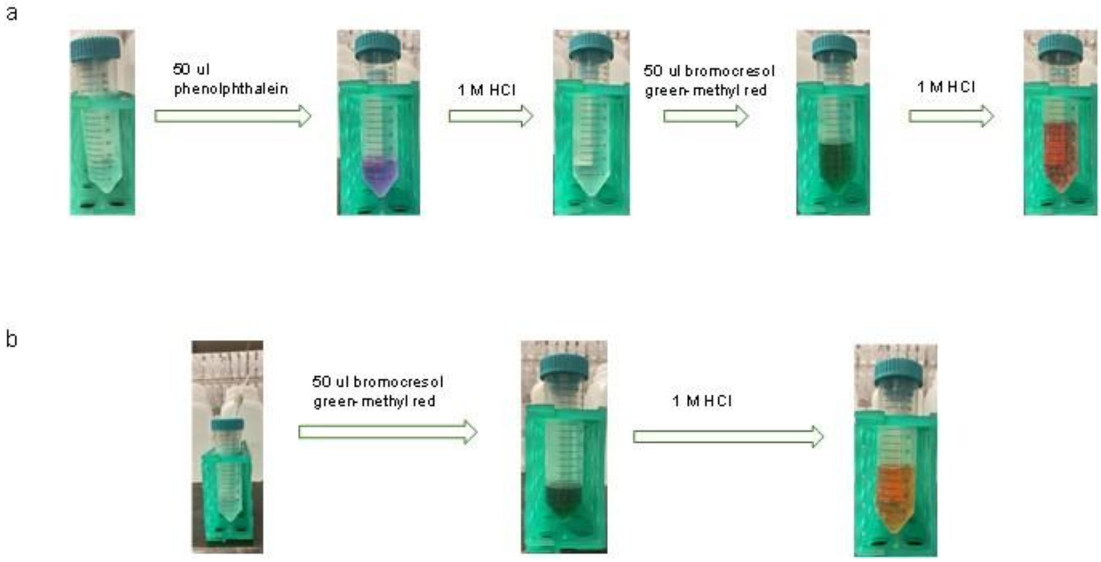

Figure S39. Device images. Color change method to detect the ability of ammonia to adsorb carbon dioxide (a) ammonia solution with KOH and (b) ammonia solution without KOH.

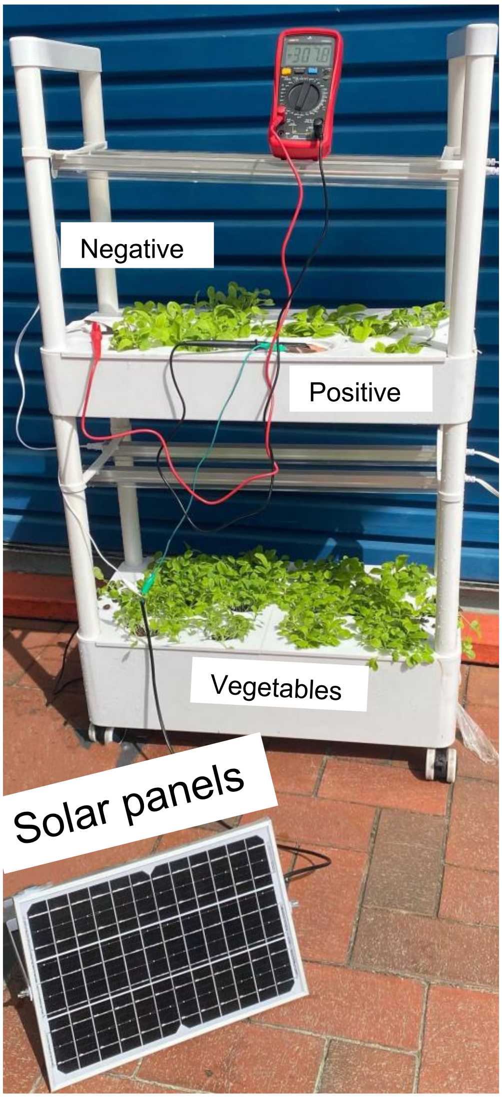

Figure S40. Schematic diagram of a commercial solar-driven ammonia generation device for plant growth.

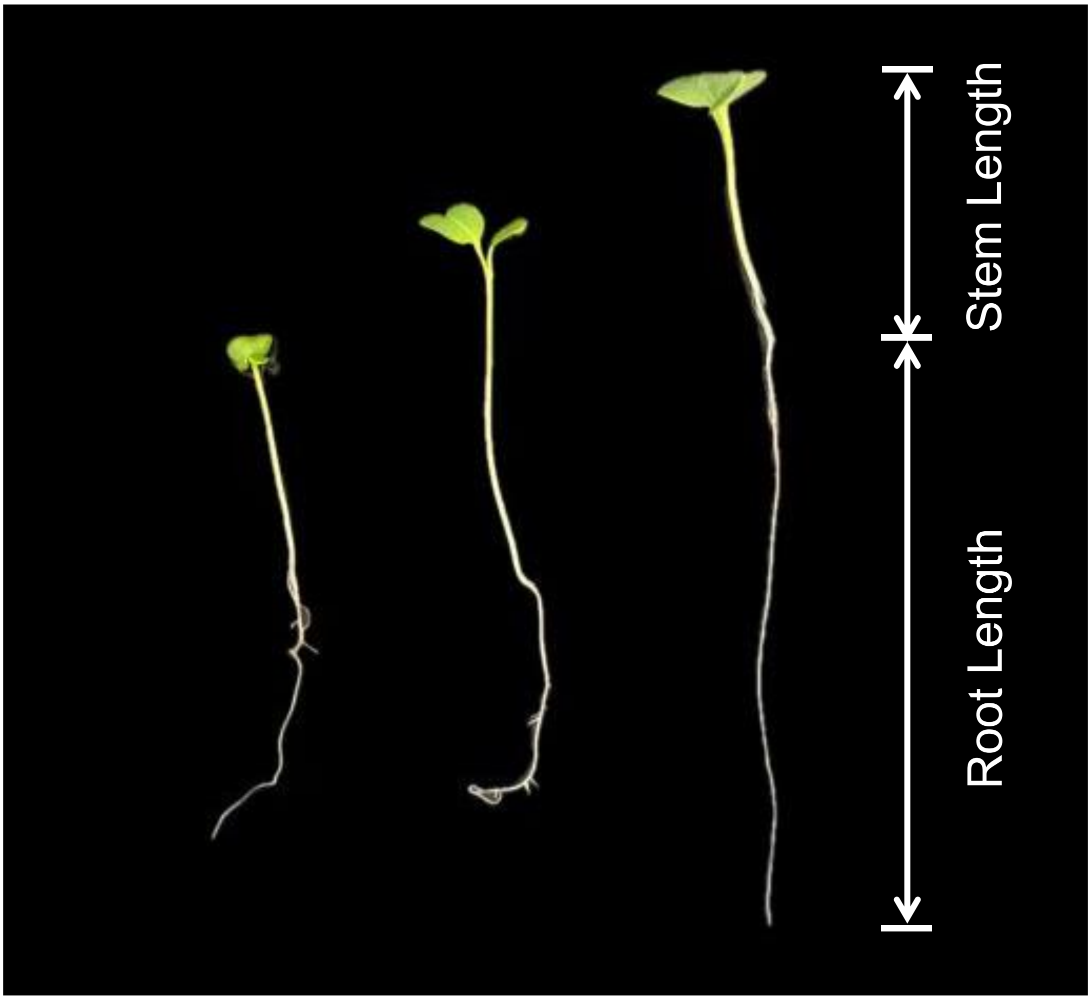

Figure S41. Schematic diagram of measuring root and stem length of plants.

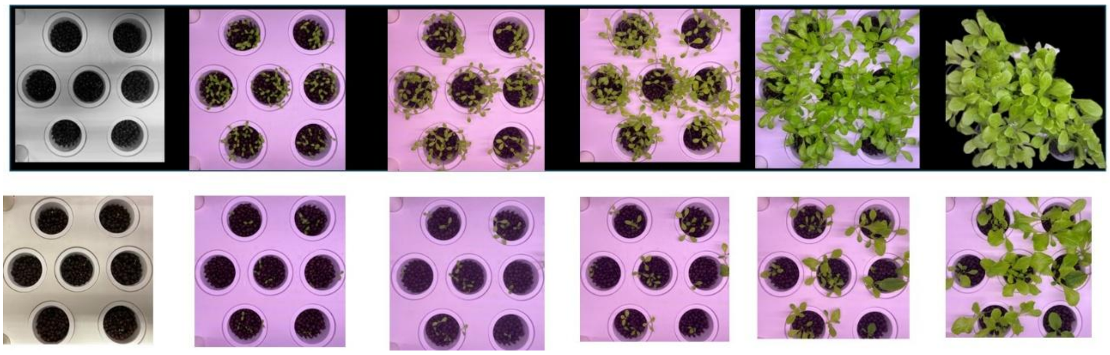

Figure S42. Comparison of lettuce growth at different stages in the STA and control groups.

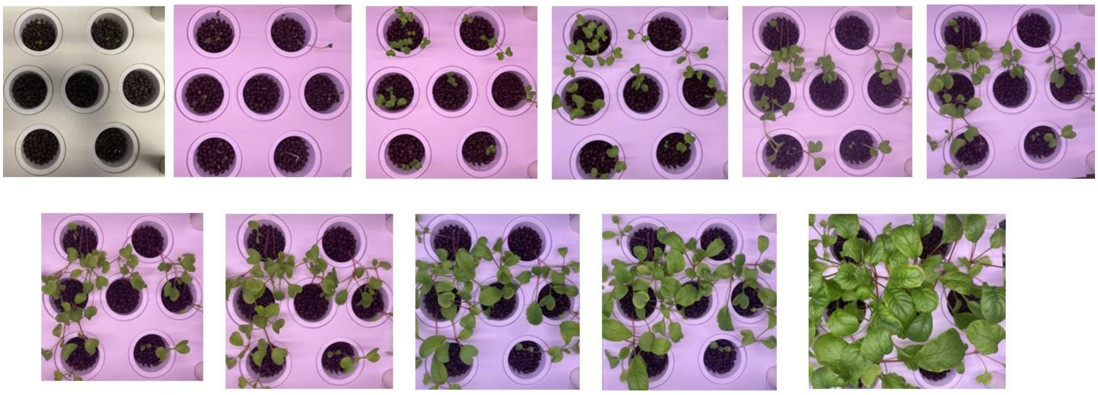

Figure S43. Comparison of cabbage growth at different stages in the STA group.

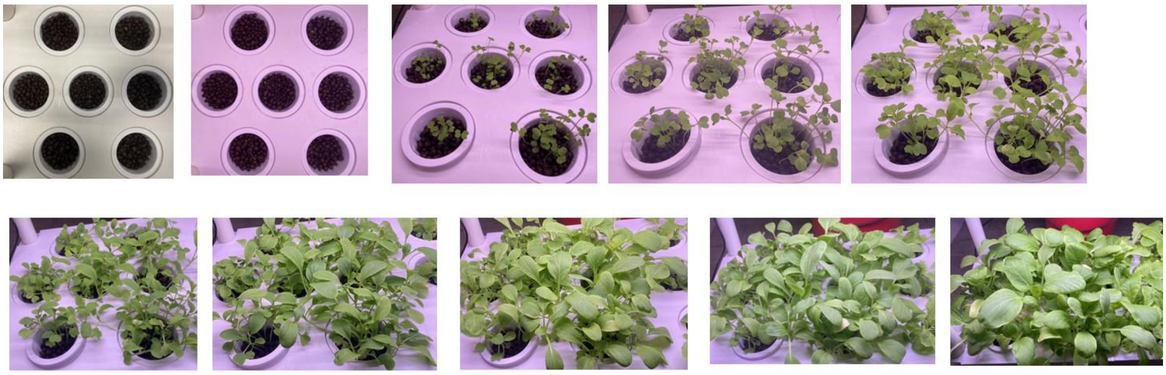

Figure S44. Comparison of campestris growth at different stages in the STA group.

Table S1. EXAFS fitting results of the Cu K-edge EXAFS for CuPd and Cu_9Pd_1, respectively.

Sample	path	CN	σ^2 (Å^2)	ΔE_0 (eV)	R (Å)	R-factor
CuPd	Cu-Cu	6	0.008	0.09	2.65	0.0103
	Cu-Pd	6	0.007	0.09	2.73	

S_0^2 is the amplitude reduction factor (0.9); CN is the coordination number; R is interatomic distance (the bond length between central atoms and surrounding coordination atoms); σ^2 is Debye-Waller factor (a measure of thermal and static disorder in absorber-scatterer distances); ΔE_0 is edge-energy shift (the difference between the zero kinetic energy value of the sample and that of the theoretical model). R factor is used to value the goodness of the fitting.

Table S2. Performance of perovskite solar cells under different solar radiation intensities.

Entry	Isc (mA)	Voc (V)	Pmax (mW)	Imax (mA)	Vmax (V)	PCE (%)
1 sun	92.67	4.62	288.91	82.55	3.5	19.26
0.9 sun	83.05	4.58	258.36	73.82	3.5	17.22
0.8 sun	72.26	4.54	226.50	62.92	3.6	15.10
0.7 sun	64.73	4.50	198.16	55.04	3.6	13.21
0.6 sun	53.75	4.46	166.94	46.37	3.6	11.13
0.5 sun	45.76	4.43	139.23	39.78	3.5	9.28
0.4 sun	36.36	4.38	109.07	30.30	3.6	7.27
0.3 sun	27.60	4.32	77.55	22.16	3.5	5.17
0.2 sun	19.28	4.24	50.77	14.93	3.4	3.38
0.1 sun	11.06	4.08	26.02	8.39	3.1	1.73

Table S3. Comparison of the NTIRR performance of the L-CuPd in this work with the previously reported Cu-based catalysts.

Catalysts	FE(%)	Current density (mA cm^{-2})	Ammonia Yield (mg/mmol h^{-1} mg_{cat}^{-1})	Stability (h)	Ref.
L-CuPd	90	600	65.01 mg h^{-1} mg_{cat}^{-1}	100h	★This work
RuCu DAs/NGA	91	700	-	24h	1
M-Cu/Cu_2O	95	180	0.29 mmol h^{-1} mg^{-1}	3h	2
FeTeSe	98.7	160	40 mg h^{-1} mg_{cat}^{-1}	5h	3
Fe/Cu-HNG	92.51%	40	1.08 mmol h^{-1} mg^{-1}	24h	4
CuPd nanocube	92.5%	310	6.25 mmol h^{-1} mg^{-1}.	12h	5
Cu–PTCDA	78%	40	0.0256 mmol h^{-1} cm^{-2}	4h	6
$Cu_{50}Co_{50}$	99%	1098	4.58 mmol h^{-1} cm^{-2}	10h	7
Pd-Cl/Cu_2O	99%	~2180	330 mg h^{-1} cm^{-2}	-	8
CoP-CNS	80	540	3.093 mmol h^{-1} cm^{-2}	120h	9

Table 4. CO_2 capture from different sources.

Condition	CO_2 Capture Rate	Primary Species	Notes
1M KOH + 1M KNO_3 (electrolysis)	18.5 mg h^{-1}	NH_4HCO_3 + K_2CO_3/$KHCO_3$	Enhanced by NH_3 production and Background KOH absorption
1M KNO_3 (electrolysis)	15.0 mg h^{-1}	NH_4HCO_3	Enhanced by NH_3 production
1M KOH （No electrolysis)	3.1 mg h^{-1}	K_2CO_3/$KHCO_3$	Background KOH absorption

Note S1

The energy efficiency of Artificial photosynthesis. The energy efficiency of biological photosynthesis is defined as the percentage of the energy content of harvestable biomass relative to the total solar irradiance energy over the same area (with solar energy defined as 100%). To compare our process with biological photosynthesis, we calculated the efficiency of converting sunlight into biomass. This process utilizes a photovoltaic system to power the electrolytic production of ammonia, which is subsequently used in hydroponic plant cultivation. We define the energy efficiency as the solar energy input minus the energy conversion losses in perovskite solar cells, minus the energy losses in the electrocatalytic reaction for ammonia production, and minus the energy losses during the plant's absorption of ammonia. The increase in the energy content of the biomass is calculated as follows:

$$\text{Artificial photosynthesis} = (100\%-P_{\text{Perovskite photovoltaic losses}}-P_{\text{Loss during NITRR}})*\text{Nitrogen absorption rate of plants}$$

(1) $P_{\text{Perovskite photovoltaic losses}}$ = 1-PCE of perovskite solar cells =1-19.26%=80.74%

19.26% comes from Supplementary Table 2 of perovskite solar cells under different solar radiation intensities.

(2) $P_{\text{Loss during NITRR}}$ = 1- FE of ammonia = 1- Average value of FE of ammonia at 0.5~1.0 solar intensity (comes from Fig. 3c) = 1-86%=14%

(3) Nitrogen absorption rate of plants=66.5%

The literature widely indicates that the Nitrogen Use Efficiency (NUE) in traditional soil-based cultivation typically ranges from 30% to 50%, whereas in hydroponic systems, particularly closed-loop recirculating systems, NUE can easily reach 70% to 90% or even higher[10,11]. To maintain a more conservative and universally applicable estimate, we selected 66.5% as the ammonia absorption rate.

Based on above discussion,

$$\text{Artificial photosynthesis} = (100\%-P_{\text{Perovskite photovoltaic losses}}-P_{\text{Loss during NITRR}})*\text{Nitrogen absorption rate of plants}$$

$$= (100\%-80.74\%-14\%) * 66.5\%= 3.5\%$$

Note S2

The benefit-cost analysis was carried out using a modified model to calculate the total cost of production with units of US$ per ha of lettuce via solar-to-NITRR to lettuce, a traditional planting of lettuce, and lettuce revenue price in 2025, respectively.

(1) For solar-to-ammonia to lettuce: The total cost includes cost of solar panels, electrolyze cost, catalyst cost and base fertilizer.

The price of electrolyzer is assumed to be 20000 $ m^{-2}, our electrolyzer is 0.3*0.3 m^2, so cost of electrolyzer is 20000*0.09=1800 $.[12]

The catalyst cost is assumed to be 10% of the electrolyzer cost[9].

The base fertilizer is assumed to be 30% of the total fertilizer, which is 496.1

The cost of solar panels is assumed to be 200 $.

Thus, the total cost for solar-to-ammonia

= 1800+180+496.1+200 = 2676.1 $ m^{-2}

(2) For traditional planting: The total cost includes fertilizer and labor cost.

The average cost of chemical fertilizer was $ 1653.6 ha^{-1}, and the labor cost for applying chemical fertilizer was $ 826.8 ha^{-1} per person. Without considering the potential cost of environmental pollution remediation, the costs of traditional fertilization methods were $ 2480.4 ha^{-1} $year^{-1}$ on average.

(3) The revenues can be calculated based on the market price of lettuce.

In terms of revenue, the average selling price of lettuce was $ 0.3 kg^{-1}, with an average yield of 37500 kg ha^{-1} and a revenue of $11250 ha^{-1}. The maximum yield using the solar-to ammonia lettuce was 52500 kg ha^{-1} (1.4 folds), and the corresponding maximum revenue was $15750 ha^{-1}.

Reference

[1] Liu, K. *et al.* Tailoring asymmetric RuCu dual-atom electrocatalyst toward ammonia synthesis from nitrate. *Nature Communications* **16**, 2167 (2025).

[2] Lu, Y. *et al.* Size-effect induced controllable Cu0-Cu+ sites for ampere-level nitrate electroreduction coupled with biomass upgrading. *Nature Communications* **16**, 2392 (2025).

[3] Liu, J. *et al.* Reaction-driven formation of anisotropic strains in FeTeSe nanosheets boosts low-concentration nitrate reduction to ammonia. *Nature Communications* **16**, 3595 (2025).

[4] Zhang, S. *et al.* Fe/Cu diatomic catalysts for electrochemical nitrate reduction to ammonia. *Nat. Commun.* **14**, 3634 (2023).

[5] Gao, Q. *et al.* Breaking adsorption-energy scaling limitations of electrocatalytic nitrate reduction on intermetallic CuPd nanocubes by machine-learned insights. *Nat. Commun.* **13**, 2338 (2022).

[6] Chen, G.-F. *et al.* Electrochemical reduction of nitrate to ammonia via direct eight-electron transfer using a copper–molecular solid catalyst. *Nat. Energy* **5**, 605-613 (2020).

[7] Fang, J. Y. *et al.* Ampere-level current density ammonia electrochemical synthesis using CuCo nanosheets simulating nitrite reductase bifunctional nature. *Nat. Commun.* **13**, 7899 (2022).

[8] Liao, W. *et al.* Sustainable conversion of alkaline nitrate to ammonia at activities greater than 2 A cm(-2). *Nat Commun* **15**, 1264 (2024).

[9] Fan, K. *et al.* Active hydrogen boosts electrochemical nitrate reduction to ammonia. *Nat. Commun.* **13**, 7958 (2022).

[10] Amirouche, M., Smadhi, D. & Zella, L. in *Nitrogen in Agriculture - Physiological, Agricultural and Ecological Aspects* (eds Takuji Ohyama & Kazuyuki Inubushi) (IntechOpen, 2020).

[11] Chamoli, N. *et al.* Comparative Analysis of Hydroponically and Soil-Grown Lettuce. *Journal of Mountain Research* **19** (2024).

[12] Krishnan, S. *et al.* Present and future cost of alkaline and PEM electrolyser stacks. *International Journal of Hydrogen Energy* **48**, 32313-32330 (2023).
